# Detection of cell-free microbial DNA using a contaminant-controlled analysis framework

**DOI:** 10.1186/s13059-021-02401-3

**Published:** 2021-06-23

**Authors:** Enrique Zozaya-Valdés, Stephen Q. Wong, Jeanette Raleigh, Athena Hatzimihalis, Sarah Ftouni, Anthony T. Papenfuss, Shahneen Sandhu, Mark A. Dawson, Sarah-Jane Dawson

**Affiliations:** 1 Peter MacCallum Cancer Centre, Melbourne, Australia; 2 Sir Peter MacCallum Department of Oncology, University of Melbourne, Melbourne, Australia; 3 Walter and Eliza Hall Institute of Medical Research, Melbourne, Australia; 4 Department of Medical Biology, University of Melbourne, Melbourne, Australia; 5 Centre for Cancer Research, University of Melbourne, Melbourne, Australia

**Keywords:** Cell-free microbial DNA, Plasma, Microbiome, Cancer, Contamination, 16S rRNA gene

## Abstract

**Background:**

The human microbiome plays an important role in cancer. Accumulating evidence indicates that commensal microbiome-derived DNA may be represented in minute quantities in the cell-free DNA of human blood and could possibly be harnessed as a new cancer biomarker. However, there has been limited use of rigorous experimental controls to account for contamination, which invariably affects low-biomass microbiome studies.

**Results:**

We apply a combination of 16S-rRNA-gene sequencing and droplet digital PCR to determine if the specific detection of cell-free microbial DNA (cfmDNA) is possible in metastatic melanoma patients. Compared to matched stool and saliva samples, the absolute concentration of cfmDNA is low but significantly above the levels detected from negative controls. The microbial community of plasma is strongly influenced by laboratory and reagent contaminants introduced during the DNA extraction and sequencing processes. Through the application of an in silico decontamination strategy including the filtering of amplicon sequence variants (ASVs) with batch dependent abundances and those with a higher prevalence in negative controls, we identify known gut commensal bacteria, such as *Faecalibacterium*, *Bacteroides* and *Ruminococcus*, and also other uncharacterised ASVs. We analyse additional plasma samples, highlighting the potential of this framework to identify differences in cfmDNA between healthy and cancer patients.

**Conclusions:**

Together, these observations indicate that plasma can harbour a low yet detectable level of cfmDNA. The results highlight the importance of accounting for contamination and provide an analytical decontamination framework to allow the accurate detection of cfmDNA for future biomarker studies in cancer and other diseases.

**Supplementary Information:**

The online version contains supplementary material available at 10.1186/s13059-021-02401-3.

## Background

The human microbiota is a major factor governing health and disease [1–3]. In cancer, the gut microbiome has emerged as having an important influence on disease progression, treatment responses and toxicity to immunotherapy [4, 5]. Several landmark studies in mouse models and melanoma patients have examined changes in the gut flora after treatment with checkpoint blockade and found that treatment responses were highly dependent on the diversity and abundance of specific bacterial species [6–9]. These and other findings suggest that characterisation of the host microbiome is critically important in order to understand and potentially manipulate responses to cancer therapies. However, a major barrier for analysing the gut microbiome as a cancer biomarker is the need for collection of stool samples. Here, there is often significant non-compliance due to the undesirable nature of this type of collection which can be challenging from a patient’s perspective [10].

It is currently unclear whether assessment of the human microbiome from other types of biospecimens, may also prove to be important cancer biomarkers. In addition to nucleic and mitochondrial sources of cell-free DNA (cfDNA) found in blood plasma, accumulating evidence indicates that commensal microbiome-derived DNA may also contribute to the composition of cfDNA [11–15]. However, to date, there has been limited use of rigorous experimental controls to account for contamination that invariably affects low-biomass microbiome studies [16–18]. Moreover, the source, type and abundance of specific bacterial DNA in plasma is not well characterised. These steps are essential before the circulating microbiome can be investigated as a potential cancer biomarker and translated into clinical use.

In this study, we sought to determine if cell-free microbial DNA (cfmDNA) from plasma could be detected using a rapid and high throughput 16S-rRNA-gene based approach, well suited to low-biomass, high-host-DNA samples. Through analysis of plasma samples from stage IV melanoma patients and healthy individuals, we assessed the influence of contamination and applied a stringent filtering strategy to address the challenges associated with the low-biomass microbiome, in order to determine if a genuine circulating cfmDNA signal could be recovered and if differences could be detected between cfmDNA from healthy individuals and cancer patients.

## Results

### Experimental design

**Fig. 1:**
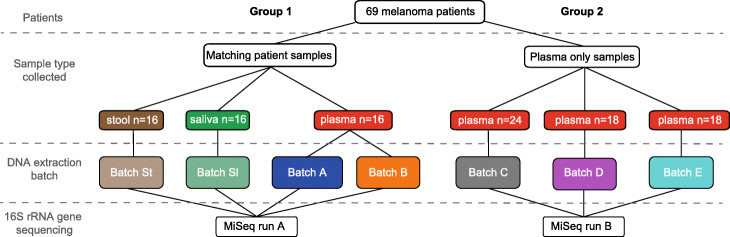
Schematic overview of the study. Biospecimens from a total of 69 stage IV melanoma patients (showing no signs of infection) were analysed across two groups: (1) with matching patient stool, saliva and plasma samples and (2) with plasma samples only. Samples were extracted for DNA in different batches totalling *n* = 1 for stool, *n* = 1 for saliva and *n* = 5 for plasma (batches A–E). Thirteen of the sixteen samples of batch A matched samples of batch B. The number of DNA-Extraction-Negative-Controls (DENCs) extracted in each one of the depicted batches from left (batch St) to right (batch E) were 12, 6, 9, 9, 8, 8 and 8 respectively. All samples were amplified for the 16S rRNA gene and sequenced across two MiSeq runs that correspond to each one of the two groups

Our study initially analysed 89 plasma samples collected from a cohort of 69 stage IV melanoma patients showing no sign of infection (Additional file 1: Table S1). The plasma samples were divided into two distinct groups (Fig. 1). The first group (samples, *n* = 16) was used to assess the influence of potential contamination when analysing low biomass microbiome samples (plasma-derived cfmDNA) versus high biomass microbiome samples such as those from the gut (stool) or the oral cavity (saliva). Hence, for each patient, we analysed temporally matched plasma, stool and saliva samples. Each plasma sample was extracted in replicate across two separate DNA-extraction-batches (hereafter referred to as DEB) for which different units of the same DNA extraction kit were used (batches A and B). A second group of plasma samples (total *n* = 60) were extracted for cfDNA across three DEBs (batches C, D, E), each of which also used different units of the same DNA extraction kit (Fig. 1). Due to sample availability, plasma samples in group 2 were not extracted in replicate and did not have temporally matched stool or saliva samples. Across both groups, every DEB included between 6 to 12 DNA Extraction Negative Controls (DENCs) where nuclease-free water was the only input used for the DNA extraction. All plasma DNA extractions were conducted by limited personnel using a disposable surgical gown and gloves in a biosafety cabinet (solely dedicated to blood handling) in which all equipment had been disinfected and DNA-cleaned (using 1% virkon, DNA decontamination reagent and UV light).

### Concentration levels of cfmDNA from plasma

**Fig. 2:**
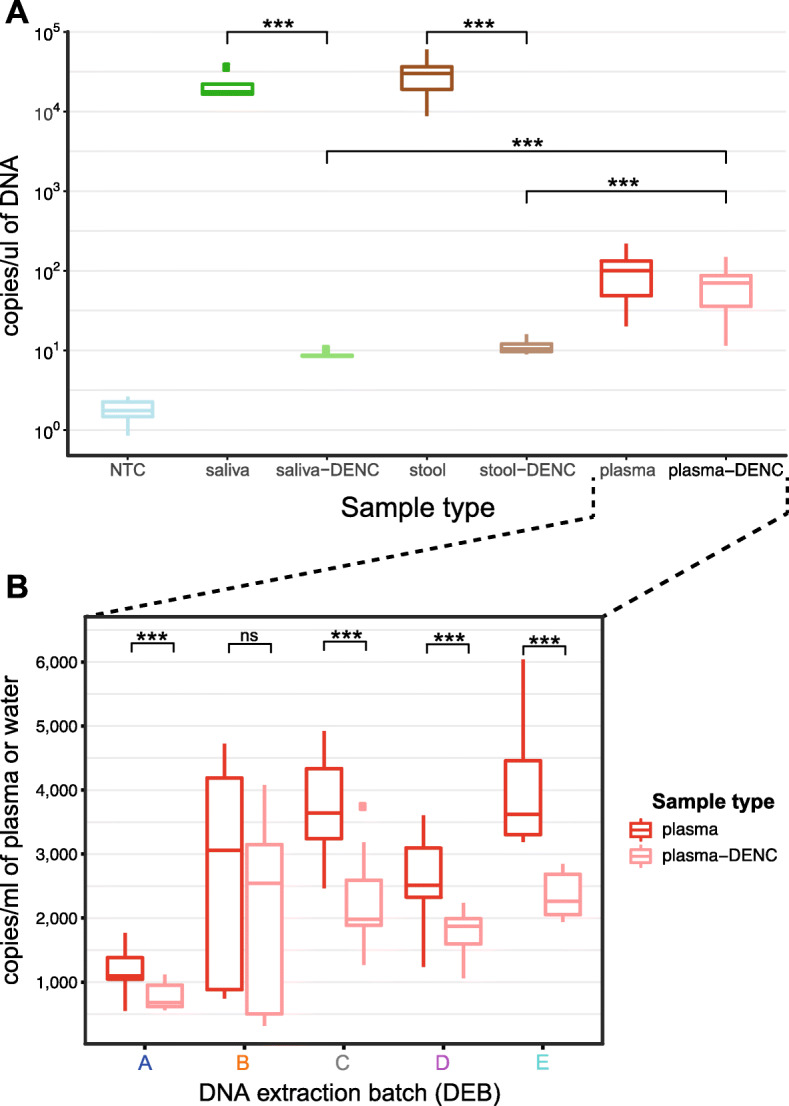
Absolute levels of bacterial DNA from saliva, stool and plasma. A droplet digital PCR (ddPCR) targeting the V4 region of the 16S rRNA gene was used to measure the absolute levels of all bacterial DNA from different biospecimen sources. **A** The levels of bacterial DNA are shown for a subset of 10 stool, 4 saliva and 58 plasma samples collected in the study and a subset of 12, 4 and 40 DNA Extraction Negative Controls (DENCs) corresponding to each sample-type, respectively. For DENCs, nuclease-free water was the only input used for the DNA extraction. Levels are also shown for 28 replicates of a non-template control (NTC) where nuclease-free water was the only input used for the ddPCR reaction. The sample to DENC median ratio for saliva, stool and plasma were 2064, 2919, and 1.43, respectively. The plasma-DENC to saliva-DENC and stool-DENC ratio were 8.23 and 6.77, respectively. **B** The concentration levels of microbial DNA in plasma are shown for each individual DNA extraction batch (DEB) together with their corresponding DENCs. The number of plasma samples tested for DEB A-E was 15, 10, 14, 11 and 8, respectively. The number of plasma-DENC replicates tested for DEB A-E was 9, 9, 8, 8 and 6 respectively. The plasma to DENC median ratio for DEBs A to E, were 1.62, 1.2, 1.84, 1.34 and 1.6, respectively. Statistical significance for differences between groups was determined by generalised estimating equations (GEE) test; *** *p* < 0.001, ns-not significant

To assess the absolute levels of microbial DNA concentration across all sample-types and corresponding DENCs, a universal 16S-rRNA-gene droplet digital PCR (ddPCR) assay was run across a subset of 72 samples and 58 DENCs (see the ‘Methods’ section). As expected, the microbial DNA concentration in plasma (median of 101 copies/μl of DNA) was much lower than the concentrations measured in stool (median of 30436 copies/μl of DNA) and saliva (median of 17710 copies/μl of DNA) (Fig. 2A). However, despite showing a much smaller sample to DENC ratio than stool and saliva, the overall microbial DNA concentration of plasma was greater than its corresponding DENCs (median of 71 copies/μl of DNA). To assess if this and other differences in concentrations were statistically significant, we applied generalised estimating equations (GEE) (see the ‘Methods’ section). For plasma, this showed a significant interaction between sample-vs-DENC and DEB (*p* value < 0.001). Therefore, differences between plasma and DENC were assessed within each DEB (Fig. 2B). A higher microbial DNA concentration of plasma-vs-DENC was observed across all DEBs, and these differences were consistent and statistically significant in all but one DEB (Fig. 2B). Across all plasma samples and DEBs, a mean of 2719 copies per ml of plasma versus a mean of 1829 copies per ml of nuclease-free water for plasma-DENCs was observed. Additionally, the microbial DNA concentration levels of plasma-DENC samples were significantly higher than those of stool and saliva DENCs suggesting that the plasma DNA extraction kit reagents contained higher levels of contaminant DNA than the stool and saliva DNA extraction kits used (Fig. 2A).

### Microbial community analysis of cfmDNA

Once we had established that cfmDNA concentrations were significantly higher than background contamination levels (i.e. DENC), we next assessed if differences could be observed at the level of the microbial community structure and how this compared with other sample types. Here, the two groups of samples from the patient cohort (Fig. 1) were subjected to separate amplicon sequencing runs covering the 16S rRNA gene V4 region on the Illumina MiSeq platform (see the ‘Methods’ section, Additional file 1: Figure S1). Quality processing and correction of amplicon sequencing read errors generated amplicon sequence variants (ASV) which allowed us to obtain the maximum phylogenetic resolution possible (see the ‘Methods’ section). From this, saliva, stool and plasma samples on average generated 25161, 34073 and 56674 reads, respectively (Additional file 1: Figure S2). Saliva DENCs, stool DENCs and plasma DENCs generated an average of 157, 1333 and 56172 reads, respectively (Additional file 1: Figure S2). To account for any bias in library preparation, a commercial mock microbial community composed of genomic DNA of 20 evenly distributed bacterial strains was included in each sequencing run. The analysis pipeline was able to recover ASVs with genus-level classifications corresponding to all but one of the strains (*Cutibacterium* or *Propionibacterium*) from the mock microbial community across the two Miseq runs (Additional file 1: Figure S3). This is consistent with previous skin microbiome surveys which have observed that sequencing of the V4 region of the 16S rRNA gene severely underrepresents the *Cutibacterium* genus [19]. Reflecting the PCR bias commonly associated with sequencing of the 16S rRNA gene, the recovered ASVs showed an uneven abundance distribution (Additional file 1: Figure S3A). However, this abundance distribution was consistent across separate library preparation and sequencing experiments (Additional file 1: Figure S3B).

**Fig. 3:**
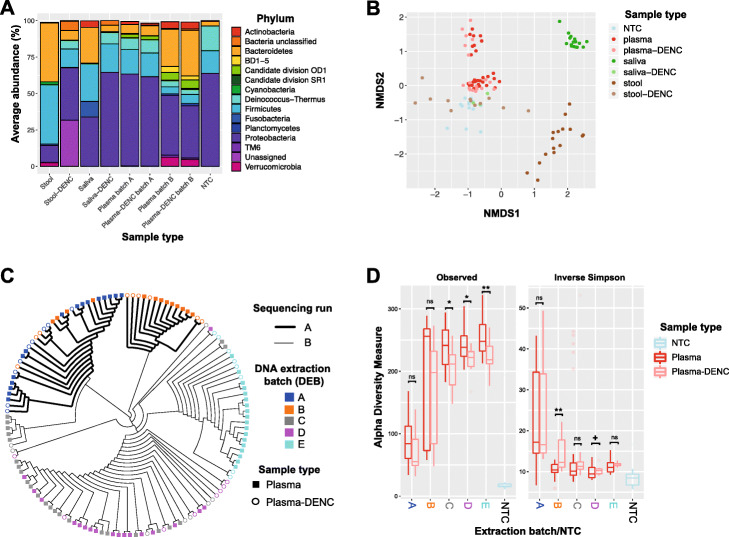
Community structure of cell-free microbial DNA in plasma and patient-matching stool and saliva. **A** Taxonomic profiles (through 16S rRNA gene sequencing) of patient-matching plasma (batches A and B only), stool and saliva samples and respective DENCs at the phylum level. The top 15 most abundant phyla are shown. **B** Non-metric multidimensional scaling (nMDS) of pairwise Bray-Curtis dissimilarities calculated from microbial community profiles for patient-matching plasma (batches A and B only), stool and saliva samples and respective DENCs at the ASV-level. **C** Dendrogram based on hierarchical clustering analysis (UPGMA) of pairwise Bray-Curtis similarities for all plasma samples (batches A–E) and their corresponding DENCs at the ASV-level highlighting batch effects due to sequencing run and DNA extraction batch (DEB). For both, **B** and **C**, prior to calculating Bray-Curtis dissimilarities, ASV counts were rarified (i.e. randomly subsampled to the minimum sample size) and square-root transformed. **D** Alpha diversity measurements of all plasma samples and their corresponding DENCs across the five DNA extraction batches A–E based on the number of observed ASVs (richness) and inverse Simpson’s Index (diversity). ‘+’ p between 0.1–0.05, * *p* < 0.05, ** *p* < 0.01, ns-not significant

Taxonomic profiling of patient-matching plasma, stool and saliva samples (group 1 in Fig. 1) showed that the microbial community structure at the phylum level differed by sample-type (Fig. 3A). As expected, stool samples were dominated by Firmicutes (av. 41%), Bacteroidetes (av. 40%), Proteobacteria (av. 12%) and Verrucomicrobia (av. 3%). In saliva, the dominant phyla were Proteobacteria (av. 34%), Firmicutes (av. 26%), Bacteroidetes (25%), Fusobacteria (av. 11%) and Actinobacteria (av. 4%). In contrast, plasma samples contained mainly Proteobacteria (av. 53%), Bacteroidetes (av. 15%), Firmicutes (av. 11%), Deinococcus-Thermus (av. 6%), Candidate division OD1 (av. 3%), Actinobacteria (av. 3%) and Verrucomicrobia (av. 3%). Further confirming the extent to which the microbiome composition differed between sample types, these differences increased with the taxonomic level depth (Additional file 1: Figure S4) and were also observed at the ASV-level (Fig. 3B). A repeated-measure aware permutational analysis of variance (RMA-PERMANOVA) of pairwise Bray-Curtis dissimilarities showed an overall significant difference by sample-type (*p* value < 0.001) and between all sample-type pairs (*p* value < 0.001 for all three pairs of sample types). Patients shared an average of 4 (0–15; 12% of ASVs and 16% of reads) plasma ASVs with stool and 1 (0–7; 6% of ASVs and 4% of reads) with saliva (Additional file 1: Table S2).

The phylum-level taxonomic profiles of stool and saliva samples were markedly different from their corresponding DENCs (Fig. 3A), and these differences increased with the taxonomic level depth (Additional file 1: Figure S4). Clear differences were also evident in the community structure at the ASV-level (Fig. 3B; ‘DNA extraction day’ adjusted PERMANOVA *p* value < 0.001 and *p* value < 0.01 for Stool vs. Stool-DENC and Saliva vs. Saliva-DENC comparisons, respectively). In contrast, there was much higher similarity in the taxonomic profiles of plasma samples and their corresponding DENCs (Fig. 3A), and this pattern was maintained along the taxonomic hierarchy (Additional file 1: Figure S4) as well as observed at the ASV-level (Fig. 3B). These results indicate that, in contrast to stool and saliva, the characterisation of the plasma microbiome is highly susceptible to contaminant DNA.

To further compare the microbial community structure of plasma and plasma-DENCs, we exclusively analysed these two sample-types across the entire patient cohort (DEB A-E) (Fig. 1 groups 1 and 2). A hierarchical clustering analysis of Bray-Curtis dissimilarities at the ASV-level showed batch effects due to sequencing run, DEB and DNA-extraction-day (Fig. 3C and Additional file 1: Figure S5) all of which were statistically significant (‘sample type’-adjusted RMA-PERMANOVA *p* value < 0.05 for sequencing run and *p* value < 0.001 for both DEB and DNA-extraction-day). The overall pattern which showed that most plasma and DENC samples from the same DEB clustered together (Fig. 3C) and the high taxonomic similarity within each DEB (Fig. 3A, Additional file 1: Figures S4 and S6) suggested that this effect, at least in part, was the consequence of contaminant DNA. The batch effect associated with DEB was especially evident by the fact that despite representing the same plasma samples, the microbial communities of most samples from DEB A and B clustered separately. Despite these batch effects, within DEBs most plasma samples clustered separately from their corresponding DENCs (Fig. 3C) and after adjusting for batch effects, this difference was statistically significant across the entire cohort (RMA-PERMANOVA *p* value < 0.05 after adjusting for sequencing-run and DEB separately and *p* value < 0.001 after adjusting for DNA-extraction-day). To account for the compositional nature of microbiome data [20], we additionally performed hierarchical clustering analysis based on Aitchison distances (i.e. Euclidean distances of clr transformed data) (Additional file 1: Figure S7). Similar to our previous results, significant batch effects were observed (‘sample type’-adjusted RMA-PERMANOVA *p* value < 0.01 for sequencing run and *p* value < 0.001 for both DEB and DNA-extraction-day). However, consistent with our previous findings, there was a significant difference between the microbiome composition of cfmDNA and negative controls (RMA-PERMANOVA *p* value < 0.05 after adjusting for sequencing run and DEB separately and *p* value < 0.001 after adjusting for DNA-extraction-day).

A generalised estimating equation (GEE) test showed that after adjusting for DEB, the overall ASV richness of plasma samples was significantly higher than DENCs (*p* value < 0.001), and this difference was significant in three of the individual DEBs (Fig. 3D). Plasma samples showed a smaller inverse Simpson index than DENCs across the cohort (GEE *p* value < 0.05). However, when comparing within individual DEBs, this difference was only statistically significant in DEB B (GEE *p* value < 0.01) (Fig. 3D). Overall, these results indicate that whilst there are significant batch effects associated with contaminant DNA, once adjusted for, a small but significant difference between the microbiome composition of cfmDNA and DENCs is observed.

### In silico decontamination identifies high-confidence plasma ASVs

**Fig. 4:**
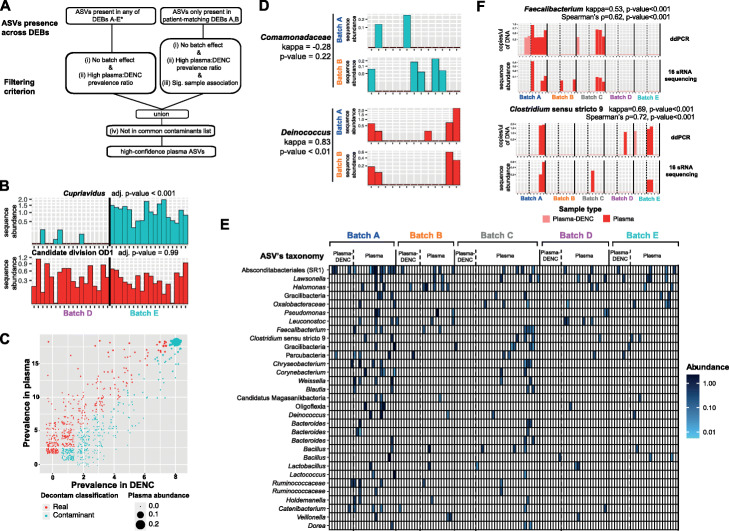
Identification of high-confidence plasma ASVs through an in silico decontamination filtering strategy. **A** Schematic description of the bioinformatics filtering strategy used to identify high-confidence plasma ASVs. *but not exclusively in A, B. **B** Representative example of an ASV not passing criterion (i) due to a significant differential enrichment in DEB E vs. DEB D (*Cupriavidus* [blue]) compared to one which passes this criterion where there is no significant abundance difference between DEBs (candidate division OD1 [red]). **C** A representative example of the filtering strategy for criterion (ii) showing the prevalence in plasma vs. DENC across all ASVs present in DEB E and the decontam [22] classification as ‘real’ or ‘contaminant’. The abundance which is represented by the size of the data points is the average relative abundance (i.e. number of reads normalised by the sample size) of an ASV across plasma samples. **D** A representative example of an ASV not passing criterion (iii) due to a lack of significant association in the detection between patient-matching plasma samples of DEBs A and B (*Comamonadaceae* [blue]) compared to an ASV which passes this criterion where there is a significant association between patient-matching samples (*Deinococcus* [red]). Cohen’s kappa inter-rater reliability coefficient was used to assess the agreement of detection between matching samples. **E** The prevalence and abundance across plasma samples and DENCs of 31 high-confidence plasma ASVs identified through using filtering criteria (i) to (iv) as described. The abundance represents a log10 transformation of the percentage of reads per plasma sample. **F** Orthogonal validation of a *Faecalibacterium* ASV and Clostridium sensu stricto 9 ASV using an ASV-specific ddPCR. Kappa coefficient was used to assess the agreement of detection between the ddPCR and 16S rRNA sequencing results. In **B**, **D** and **F**, the sequence abundance represents the percentage of reads per sample and across the upper and lower panels; each bar corresponds to the same plasma or plasma-DENC sample

To identify genuine ASVs from plasma, we developed a strategy to specifically remove contaminant DNA sequences from our dataset. Previous studies have shown that in order to separate true signal from contaminant DNA in low-biomass samples, a combination of bioinformatics techniques must be applied [18, 21]. Due to the high levels of contaminant DNA affecting our cfmDNA analysis that we had identified, we used a conservative approach and applied three bioinformatics decontamination criteria that an ASV must meet in order to be considered as a high-confidence plasma ASV (Fig. 4A): (i) no significant differential abundance due to technical variables that cause batch effects (i.e. sequencing run, DEB and DNA-extraction-day) as assessed with the limma R package [23], (ii) a higher prevalence in plasma vs. plasma-DENC samples across DEBs as assessed with the decontam R package [22] and (iii) for ASVs present only in patient-matching DEBs A and B, to have significant association in detection of the ASV between replicates using an inter-rater reliability kappa score (*p* value < 0.05 and kappa > 0.4). Representative examples of ASVs passing or not passing each of these filtering criteria are shown in Fig. 4B–D. For instance, Fig. 4C shows a representative example of the prevalence in plasma vs. DENC across all ASVs present in DEB E and the decontam classification as ‘real’ or ‘contaminant’. Similar results were observed for the other DEBs (Additional file 1: Figure S8).

A summary of the number of ASVs that met each criterion separately and in combination are shown in Table 1. Out of a total of 1506 ASVs present across all plasma samples, 239 (16%) passed criterion (i), and of these, only 8 and 4 were present across plasma samples in medium (0.1–1%) and high abundances (> 1%), respectively. A total of 329 ASVs (22%) met criterion (ii), and of these, 16 had medium abundances. Showing a significant association of detection between patient-matching samples, 20 ASVs (1.3%) met criterion (iii) and of these, 6 had medium abundances. After applying criterion (i) and (ii) to all ASVs, and criterion (iii) to ASVs only present in patient-matching DEBs A and B, a list of 38 low-abundance (< 0.01%) ASVs was obtained. As a final decontamination step (criterion (iv)), this list of ASVs was compared against a previously published ‘contaminants-blocklist ’[16] which compiles a list of taxa commonly found in laboratory reagents (e.g. DNA extraction kit buffers) that have been reported as contaminants in multiple low-biomass microbiome studies. The literature was also searched for evidence of the ASVs’ taxonomic affiliations being identified as a commensal or human pathogen. Supporting our bioinformatics decontamination strategy, only 7/38 (18%) ASVs had no evidence supporting their role as a commensal or pathogen, and of these, five were present in the ‘contaminants blocklist’. These were removed following the final decontamination step, resulting in a final list of 31 high-confidence plasma ASVs that were likely to be genuine cfmDNA members (Fig. 4E and Additional file 2: Table S3).

**Table 1:** Number of plasma ASVs that met each decontamination criterion separately and in combination

	All ASVs (%)	Low abundance ASVs (%)	Med. abundance ASVs (%)	High abundance ASVs (%)
Total	1506 (100)	1384 (100)	110 (100)	12 (100)
**Criterion (i)**				
No batch effect by sequencing run	612 (40.64)	582 (42.05)	25 (22.73)	5 (41.67)
No batch effect by DEB	277 (18.39)	260 (18.79)	12 (10.91)	5 (41.67)
No batch effect by DNA ext. day	1113 (73.9)	1069 (77.24)	38 (34.55)	6 (50)
No batch effect by any tech. var.	239 (15.87)	227 (16.4)	8 (7.27)	4 (33.33)
**Criterion (ii)**				
Decontam in DEB A	183 (12.15)	144 (10.4)	36 (32.73)	3 (25)
Decontam in DEB B	364 (24.17)	307 (22.18)	51 (46.36)	6 (50)
Decontam in DEB C	573 (38.05)	504 (36.42)	68 (61.82)	1 (8.33)
Decontam in DEB D	431 (28.62)	385 (27.82)	45 (40.91)	1 (8.33)
Decontam in DEB E	452 (30.01)	395 (28.54)	55 (50)	2 (16.67)
Decontam across DEBs	329 (21.85)	313 (22.62)	16 (14.55)	0 (0)
**Criterion (iii)**				
Sample association	20 (1.33)	14 (1.01)	6 (5.45)	0 (0)
Complete bioinformatics decontamination strategy	38 (2.52)	38 (2.75)	0 (0)	0 (0)
Final list after literature-based filter	31 (2.06)	31 (2.24)	0 (0)	0 (0)

Low abundance, < 0.1%; med abundance, 0.1–1%; high abundance, > 1%; DNA ext. day, DNA extraction day; tech. var., technical variable

To orthogonally validate our 16 s rRNA gene sequencing results, two high-confidence plasma ASVs that were classified as *Faecalibacterium* and *Clostridium* sensu stricto 9 were selected for ASV-specific ddPCR, based on their average abundance across the cohort (see the ‘Methods’ section). Calculation of the inter-rater reliability kappa score and Spearman’s correlation indicated good agreement between the ddPCR and the 16S rRNA gene sequencing in detecting these ASVs (Fig. 4F). These specific ASVs were measured at a minimum of 2 × 10^−1^ copies/μl of DNA via ddPCR, within the established detection limit of the 16S rRNA sequencing assay (Additional file 1: Figure S1).

Most of the high-confidence plasma ASVs (*n* = 25) could be classified at the family or genus level, and for all of these, we found evidence in the literature for them to be commensals or pathogens (Additional file 2: Table S3). The remaining six high-confidence plasma ASVs could only be classified at high taxonomic levels (i.e. order or above), and therefore these ASVs could potentially represent novel or poorly characterised bacterial members of the human microbiome. Covering a large phylogenetic diversity, the high-confidence plasma ASVs belonged to the phyla Firmicutes (48%), Patescibacteria group (16%), Bacteroidetes (13%), Proteobacteria (10%), Actinobacteria (6%), Bdellovibrionota (3%) and Deinococcus-Thermus (3%). Interestingly, the taxonomies of 18/31 (58%) of the high-confidence ASVs obtained from this cohort were also identified as genuine cfmDNA in another recent study using a whole-metagenome sequencing approach (Additional file 2: Table S3) [15].

We also examined the overlap between the high-confidence plasma ASVs and those identified in stool and saliva samples. Eleven (35%) high-confidence plasma ASVs were also present in stool across a range of 2 (13%) to 12 (80%) of the patients (Additional file 2: Table S3). Most of these ASVs were classified into genera of bacteria commonly found in the gut such as *Faecalibacterium*, *Bacteroides* and *Ruminococcus*. Only one high-confidence plasma ASV of the genus *Veillonella* was also present in saliva samples. This ASV was present in saliva from all 15 patients and was also present in stool samples across three patients (Additional file 2: Table S3). Although determining the origin of cfmDNA was beyond the scope of the present study, these observations suggest that the gut microbiome may represent a significant source of cfmDNA.

### Identification of cfmDNA in healthy individuals versus cancer patients

**Fig. 5:**
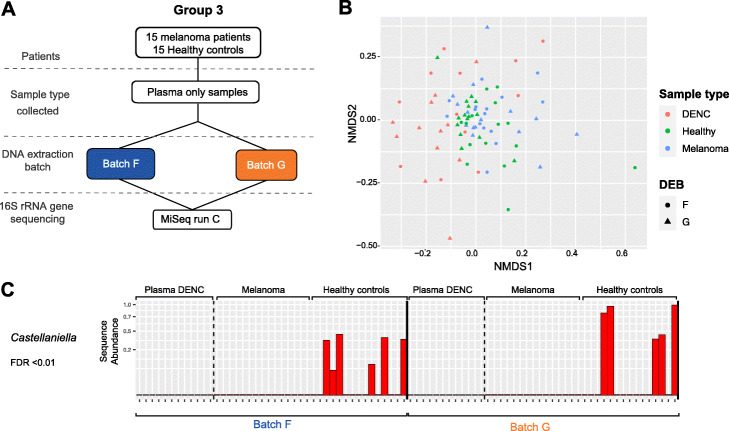
Sequencing analysis of an extension cohort of healthy individuals and melanoma patients. **A** Schematic of the experimental design used in an extension cohort of plasma samples from stage IV melanoma patients and healthy individuals. **B** Non-metric multidimensional scaling (nMDS) of pairwise Bray-Curtis dissimilarities calculated from microbial community profiles of plasma (batches F and G) and respective DENCs at the ASV-level. **C** The abundance across plasma samples and DENCs of an ASV that passed all the bioinformatics decontamination criteria (with kappa = 0.667, *p* value < 0.001) and that was classified into the *Castellaniella* genus. A significant differential abundance was observed between melanoma vs. healthy control plasma samples (FDR < 0.01) based on analysis with limma

We then assessed whether our current framework could be utilised to identify high-confidence ASVs from plasma of healthy individuals in addition to late stage cancer patients, thus providing an opportunity to compare the cfmDNA profile between these groups. We applied our 16S RNA gene sequencing protocol and analysis pipeline to a new extension cohort of stage IV melanoma patients (*n* = 15) (Additional file 1: Table S4) and healthy controls (*n* = 15) (Fig. 5A) to allow direct comparison. Here, melanoma and healthy plasma samples were extracted across two DEBs (F and G) with the same samples extracted in each batch. Each DEB consisted of three extraction runs (5 melanoma and 5 healthy controls) and included 4 DENCs. All samples were run on a single MiSeq run (Fig. 5A).

Sequencing of the same mock microbial community used in the initial cohort generated results consistent with our previous observations (Additional file 1: Figure S9A). After quality processing and generation of ASVs, healthy plasma, melanoma plasma and plasma DENC on average generated 27630, 26394 and 27679 reads, respectively (Additional file 1: Figure S9B). Taxonomic profiling of plasma samples showed a microbiome composition similar to that of the first cohort and exhibited a high similarity between plasma and plasma DENC for both melanoma and healthy samples (Additional file 1: Figure S9C). This indicates that plasma samples, regardless of belonging to melanoma patients or healthy individuals, are highly susceptible to contaminant DNA.

Consistent with the Bray-Curtis dissimilarity analysis at the ASV-level of the first cohort, ‘sample type’-adjusted RMA-PERMANOVA tests showed evidence of batch effects by DEB and DNA-extraction-run (Additional file 1: Table S5). However, in contrast to the initial cohort, here the effect size that these technical variables had on the community structure was smaller (Fig. 5B). In keeping with this smaller effect size, compositional analysis using PERMANOVA tests based on Aitchison distances showed no statistically significant differences by DEB or by DNA-extraction-run (Additional file 1: Table S5). In line with our observations in the initial cohort, Bray-Curtis dissimilarity analysis showed a degree of separation between plasma and DENC samples (Fig. 5B) and after adjusting for batch effects, PERMANOVA tests indicated that this difference was statistically significant, both for all plasma samples analysed together and for healthy and melanoma samples analysed separately (Additional file 1: Table S5). PERMANOVA tests based on Aitchison distances showed the same results (Additional file 1: Table S5). In contrast to the first cohort, batch effect-adjusted GEE tests showed no statistically significant difference in the ASV richness of plasma and DENCs (Additional file 1: Figure S9D and Table S5). However, consistent with the first cohort, plasma samples showed a statistically significant smaller inverse Simpson index than DENCs, and this could be observed both for all plasma samples analysed together and for melanoma and healthy samples analysed separately (Additional file 1: Figure S9D and Table S5). Overall, these results show that the same batch effects associated with contaminant DNA can be present when analysing plasma samples from healthy individuals; however, once adjusted for, a small but significant difference between the microbiome composition of cfmDNA from healthy individuals and DENCs is observed.

Comparison of the community structure of healthy vs. melanoma samples showed no observable nor statistically significant differences (Fig. 5B and Additional file 1: Table S5). Whilst no statistically significant difference was observed for ASV richness, a DNA-extraction-run adjusted GEE test using the inverse Simpson index indicated that plasma from healthy individuals had a higher diversity than plasma from melanoma patients (Additional file 1: Figure S9D and Table S5).

To identify high-confidence plasma ASVs from the extension cohort, we applied our in silico decontamination framework (Additional file 1: Table S6). Consistent with the smaller batch effects observed in this data set, likely contributed to by the smaller sample size and fewer DEBs, the majority of ASVs (1674/1675 (99.9%)) had no significant batch effects and therefore passed criterion (i). A total of 347 (21%) ASVs passed criterion (ii) demonstrating a significantly higher prevalence in plasma vs. DENC samples (Additional file 1: Figure S9E) and 38 (2.3%) ASVs passed criterion (iii) exhibiting a significant association of detection between patient-matching samples across DEBs. A total of six ASVs passed all three criteria (Additional file 1: Figure S9F), and of these, two passed the literature-based criterion (iv) (Additional file 3: Table S7). The genera of two of these six ASVs were identified as genuine cfmDNA in a recent study using a whole-metagenome sequencing approach (Additional file 3: Table S7) [15].

The six high-confidence ASVs that passed criteria (i) to (iii) were not part of the list of 31 high-confidence ASVs found in the first cohort. However, three of the ASVs were classified into the Gracilibacteria class, *Weeksellaceae* family and *Deinococcus* genus and ASVs belonging to the same taxa were part of the high-confidence list of the first cohort (Additional file 3: Table S7). Of the list of 347 ASVs that passed criteria (i) and (ii), three ASVs classified as Absconditabacteriales (SR1), *Oxalobacteraceae* and *Blautia* were part of the 31 high-confidence ASVs found in the initial cohort. ASVs belonging to the *Pseudomonas*, *Deinococcus*, *Bacteroides*, *Chryseobacterium*, *Bacillus*, *Lactococcus*, *Clostridium* senso stricto 9 and *Blautia* genera, as well as the *Oxalobacteraceae* family, were represented in both lists of ASVs.

To identify any differentially abundant ASVs between healthy and melanoma samples, we applied limma hypothesis testing on the set of 347 ASVs that met criteria (i) and (ii). Using linear models that separately adjusted for DEB and DNA-extraction-run, only one differentially abundant ASV was found after correcting for multiple testing (Fig. 5C). This ASV was only present in healthy individuals and was absent from any of the plasma DENC samples. It was classified into the *Castellaniella*, and although we could not find any evidence in the literature indicating that this genus could represent a human commensal or pathogen from analysis of high-biomass samples, it passed all three in silico decontamination criteria and was recently identified as a genuine cfmDNA member [15] (Additional file 3: Table S7).

## Discussion

In recent years, the role of the microbiome in cancer has been increasingly recognised, including its influence on response and toxicity to immunotherapy [5, 8, 9]. It is therefore not surprising that interest in the development of microbiome-based cancer biomarkers has grown [15, 24, 25]. Most of the evidence characterising the microbiome in cancer has come from the study of the gut microbiome. Currently, there is limited understanding of cell-free microbial nucleic acids and if these could be valuable for clinical application. Here, we examined whether a 16S rRNA gene sequencing approach of blood plasma DNA could be used to detect a genuine cfmDNA signal. From this analysis, we found that due to its low-biomass, the characterisation of cfmDNA in plasma from cancer patients and healthy individuals can be heavily affected by contaminant DNA which can produce large batch effects in the microbial diversity and community structure. However, once the variation produced by these batch effects is adjusted for, we found a small but significantly higher bacterial concentration in plasma compared to controls and a significant difference between the community structures of these two. Furthermore, we were able to leverage our experimental design to identify high-confidence plasma ASVs. Together, these observations indicate that plasma from healthy individuals and cancer patients can harbour a low yet detectable level of cfmDNA and that this framework can be utilised to identify potential differences in cfmDNA between healthy and cancer patients.

Our findings support those of several recent studies which have analysed the presence of cell-free microbial nucleic acids from plasma. Recent studies that have explored plasma cfmDNA through whole-genome sequencing include those of Kowarsky et al. [12] who reported large numbers of previously unidentified members of the human microbiome and that of Huang et al. [11] who found differences in the diversity and abundance of certain taxa between healthy females and those with early-onset breast cancer. However, these studies did not report stringent decontamination analyses and the impact that contamination can have in microbiome studies, particularly those with low endogenous biomass, such as plasma, has been widely reported in the literature [16, 21, 26–29]. Whilst our findings and those of previous studies support the existence of cfmDNA in plasma, our observations suggest that the levels and diversity are considerably lower than previously thought, once the influence of contamination is addressed. Due to the ubiquitous presence of microbes, sources of contaminant DNA include the laboratory environment, laboratory reagents and experimental procedures, as well as the researchers themselves [26, 27, 29–31]. In particular, widespread contaminant DNA in laboratory reagents and kits has led some researchers to refer to this as the ‘kitome’, which has been seen consistently across different laboratories [26]. A recent study by Poore et al. [15] was the first to apply strict decontamination strategies when analysing plasma-derived cfmDNA. By performing whole metagenome sequencing analysis of cfDNA from plasma along with contamination-controls, they were able to validate distinct cancer-type specific cfmDNA signatures that were identified by analysing a large collection of tissue and blood samples. This highlights the potential of cfmDNA based diagnostics in cancer, when appropriate decontamination strategies are applied.

Here, we implemented a framework that can be utilised for the analysis of cfmDNA which adopts guidelines to address the impact of contamination [16, 32]. Our approach included (1) modification of DNA extraction procedures to mitigate contamination, (2) inclusion of appropriate negative controls with each DNA extraction and sequencing library preparation, (3) analysis of the levels and types of contaminant taxa in negative controls via 16S rRNA gene based ddPCR and sequencing, (4) assessment and adjustment for batch effects associated with technical variables and (5) the application of in silico decontamination criteria to filter out contaminating sequences and identify high-confidence plasma ASVs. Future analyses of additional data sets and/or simulated microbial communities could help refine the in silico decontamination criteria to further optimise accuracy and sensitivity. We elected to perform 16S rRNA gene sequencing rather than whole metagenome analysis in our study. Whilst previous studies have utilised whole-genome sequencing and showed positive detection of circulating bacterial DNA from plasma, this can be an inefficient approach as only a minute fraction of total sequencing reads come from circulating bacterial DNA [12, 15]. In contrast, 16S rRNA gene sequencing specifically targets microbial DNA which makes it an option better suited for samples with low microbial biomass such as plasma. However, analysis of the 16S rRNA gene limits investigation to bacteria whose 16S rRNA gene subfragment has been characterised and may not accurately represent the whole range of microbial DNA that may be detected in the circulation [12, 15].

Our results have major implications for the analysis of cell-free microbial DNA and its potential clinical applications. Ideally, ultraclean cell-free DNA extraction kits should be developed that minimise the amount of contaminant DNA introduced into the analysis, as has been done for other sample types such as whole blood. This is supported by our results, where we found that DNA contamination levels in the cfDNA extraction kit were much higher than in extraction kits designed for microbiome analysis of stool and saliva samples. Coupling new kits with workflows that can minimise external sources of contaminant DNA and account for technical variability could significantly reduce the batch effects and allow a more accurate dissection of real signal from noise. Using this optimised workflow, further analyses with larger cohorts will be needed to identify and validate distinct ASVs associated with cancer versus healthy individuals and determine if cfmDNA can be utilised as a biomarker for clinical management.

## Conclusions

Our results serve to highlight the challenges and caveats, yet future promise of cfmDNA analysis. Despite high levels of contaminant DNA, our evidence suggests that genuine cell-free microbial DNA exists in plasma, but this can only be accurately determined when stringent decontamination analyses are applied. Future developments and refinements in laboratory and bioinformatics practices will be necessary in order for it to be exploited as a clinically viable minimally invasive biomarker for cancer and other diseases.

## Methods

### Clinical cohort and specimen collection

All plasma, stool and saliva samples were collected from stage IV melanoma patients as part of the Melanoma Biomarkers Study at the Peter MacCallum Cancer Centre in Melbourne, Victoria. Healthy control plasma was collected from donors through the Victorian Cancer Biobank (Study ID VCB_ 19014). Blood was collected in EDTA tubes and processed within 2 h of collection. Whole blood was first centrifuged at 1600*g* for 10 min to separate the plasma from the peripheral blood cells, followed by a further centrifugation step at 20,000*g* for 10 min to pellet any remaining cells and/or debris. The plasma was then stored at − 80 °C until plasma DNA extraction. Stool samples were collected into 1× OMNIgene®•GUT (OMR-200) tubes as per manufacturer’s protocol. These were stored at room temperature for delivery to the testing laboratory and stored for up to 2 months. Saliva samples were collected into 1× Oragene® Saliva Collection Kit (DNA Genotek, OG500) tubes as per manufacturer’s protocol.

### DNA extraction and extraction controls

Plasma DNA was extracted from up to 2 ml of plasma using the QIAmp Circulating Nucleic Acid Kit (Qiagen) according to the manufacturer’s instructions. All plasma DNA extractions were performed under sterile conditions (using disposable surgical gown and gloves) in a Biosafety Level 2 cabinet dedicated only to plasma DNA extractions in which all equipment had been disinfected and DNA-cleaned using 70% ethanol, 1% Virkon, DNA decontamination reagent (Sigma LookOut DNA Erase) and UV light for a minimum of 30 min. The plasma DNA was eluted into 50 μl buffer AVE and stored at − 20 °C. Faecal specimens were extracted for DNA using the MoBio PowerFecal DNA isolation kit (Qiagen) according to the manufacturer’s instructions and stored at − 20 °C. Saliva samples were extracted for DNA using the Qiasymphony SP and the QIAsymphony DSP DNA Kit (Qiagen). DNA Extraction Negative Controls (DENC) were employed where nuclease-free water (Promega) was the only input performed in each extraction to assess for possible contamination arising from the different extraction kits. *E. coli* derived DNA was extracted using the DNeasy Blood and Tissue Kit (Qiagen) according to the manufacturer’s protocols, quantified using the Qubit high-sensitivity dsDNA kit (Thermo Fisher Scientific) and diluted in nuclease-free water for the dilution series.

### 16S rRNA gene sequencing

Based on the 16S rRNA gene Illumina amplicon protocol of the Earth Microbiome Project, the V4 hypervariable region of the bacterial 16S rRNA marker gene (16Sv4) was targeted for PCR amplification [33, 34]. This PCR protocol has been used multiple times for the study of a large variety of environmental and human microbiomes [35]. Primers 515F-OH1 (ACACTGACGACATGGTTCTACAGGACTACNVGGGTWTCTAAT) and 806R-OH2 (TACGGTAGCAGAGACTTGGTCTGTGYCAGCMGCCGCGGTAA) were used, which included consensus sequences (underlined) to provide a target for the subsequent introduction of Illumina sequencing adaptors and Fluidigm index barcodes to the amplicon target for paired-end sequencing on the Illumina MiSeq instrument [36–38]. Primary 16S rRNA gene PCR amplification was performed in duplicate using the Platinum Hot-Start PCR Master Mix (2X) (ThermoFisher Scientific) with the following conditions: 94 °C for 3 min followed by 25 cycles for stool and saliva samples and 30 cycles for plasma at 94 °C for 45 s each, 55 °C for 1 min, and 72 °C for 1 min and a final extension step at 72 °C for 10 min. Amplicons from the primary amplification were then diluted 1/10 and used as template for the secondary amplification. In the secondary amplification, the overhang sequences were used to introduce Illumina sequencing adaptors and Fluidigm index barcodes to the amplicon target. Following amplification, products were pooled together, purified using AMPure XP beads and quantified using an Agilent D1000 screentape (Agilent Technologies). The indexed pool was diluted to 6pM and sequenced with the MiSeq system (Illumina) using paired end 600-cycle (2 × 311) kit. A mock bacterial community control (20 Strain Even Mix Genomic Material-ATCC® MSA-1002 T) was included in each PCR amplification run to assess for uniformity in PCR amplification. Non-template controls were also included in each run to assess for DNA contamination during the PCR amplification process.

To determine the limit of detection of the sequencing approach, this was applied to a ten-fold dilution series of *E. coli* genomic DNA ranging from 10^4^ to 10^−2^ genome copies/μl of DNA (Additional file 1: Figure S1). This showed that starting from a concentration of 10^2^ genome copies/μl of DNA, the total number of reads decreased with increasing dilutions and that *E. coli* sequences were detectable at the lowest concentration of 10^−2^ genome copies/μl of DNA which recovered 336 *E. coli* reads. As shown in previous studies, the increasing number of microbial DNA serial dilutions was correlated with the diversity (and proportion) of contaminant ASVs sequenced [26, 39].

### Sequence processing

The demultiplexed 16S rRNA gene amplicon sequences from each of the three sequencing runs were separately processed using the QIIME2 suite (version 2018.11.0) [40]. After trimming of PCR primer sequences using ‘qiime cutadapt trim-paired’ with default parameters, ‘qiime dada2 denoise-paired’ with --p-trunc-len-f 246 and --p-trunc-len-r 213 was used to generate an Amplicon Sequence Variant (ASV) per sample counts table. Using ‘qiime feature-classifier classify-sklearn’ and the Silva database (119 SSU Ref NR 99 515F/806R release) [41], ASV representative sequences were taxonomically classified. QIIME2 artefacts corresponding to the ASV per sample count table, representative sequences and taxonomic classification from each of the sequencing runs were merged and exported for downstream analyses.

### Comparative diversity and statistical analyses

Files containing an ASVs per sample count table, representative sequences and a taxonomic profile covering the complete sample collection were imported into R using the Phyloseq package (version 1.28.0) [42]. ASVs taxonomically classified as eukaryota, mitochondria or chloroplast were removed. Sequencing PCR replicates from the same biological sample (e.g. plasma DNA sample from a specific patient extracted on a certain date) were merged by summing all counts together. ASVs with an abundance across samples of the same sample type below 0.01% were removed. Previous to every comparative diversity analysis, samples were normalised by rarefying (i.e. subsampling without replacement) to a minimum sample size (i.e. number of reads) that varied depending on the sizes of the samples being compared.

For the alpha diversity analysis, richness (number of observed ASVs) and inverse Simpson values and plots were obtained using the estimate_richness and plot_richness functions of phyloseq, respectively. To test for differences in alpha diversity between plasma and plasma-DENC, generalised estimating equations (GEE) were applied using the geeglm function from the geepack R package (version 1.3-1) [43] with the family parameter set to default ‘Gaussian’. This function accounts for any potential correlation between repeated measurements as in the patient-matching plasma samples of the DNA-extraction-batches (DEBs) A and B. The regression model included the terms ‘sample type’, DEB and their interaction term (‘sample type’ x DEB) to test if differences in alpha diversity between plasma and plasma-DENC changed between DEBs.

Beta diversity analysis was performed by using the ‘distance’ function of phyloseq with method = ‘bray’ which calculates the Bray-Curtis dissimilarity of community structure between all pairs of samples. Bray-Curtis-based UPGMA hierarchical clustering and non-metric multidimensional scaling analyses were performed using the hclust (with method = ‘average’) function from the R stats package and the ordinate function (with method = ‘NMDS’ and distance = ‘bray’) from phyloseq, respectively. The count data used for both visualisation methods was square-root transformed. Because the quantity of reads obtained in any microbiome study is an arbitrary number imposed by the sequencing process, microbiome count data are in fact compositions for which the abundance of each component depends on all the other components [20, 44]. To account for the compositional nature of the data and to compare against the Bray-Curtis based analysis, the Aitchison distance between samples was also calculated. For this, an offset of ‘1’ was added to the non-rarified and non-transformed ASV count data to then be centred log ratio transformed (CLR) by using the clr function of the composition R package. To obtain the Aitchison distance, the Euclidean distance was then applied. The circular UPGMA hierarchical clustering dendogram of plasma and plasma-DENC samples was obtained by modifying the R output using Dendroscope (version 3.7.2) [45]. To assess for statistical significance between the community structure of different groups of samples with no repeated measurements (e.g. stool vs. stool-DENC), the adonis function from the vegan R package (version 2.5-6) [46] was used to perform a PERMANOVA (permutational analysis of variance) test based on Bray-Curtis dissimilarities or Aitchison distance. When samples belonged to the same patient (e.g. samples from different sample types (i.e. plasma, stool and saliva) or plasma samples extracted in replicate (i.e. across DEBs A/B and DEBs F/G), a previously published function called PERMANOVA_repeated_measures which is a modified version of adonis that implements a repeated measurement aware PERMANOVA (RMA-PERMANOVA) was used [47]. To account for repeated measurements, this function performs permutations blocked within subject. For the overall and pairwise comparison between sample types (i.e. plasma, stool and saliva), only the factor ‘sample type’ was included in the statistical model. To adjust for the potential variation due to ‘DNA extraction day’, this factor along with ‘sample type’ and the interaction (‘DNA extraction day’ x ‘sample type’) was included in stool vs. stool-DENC, saliva vs. saliva-DENC and plasma vs. plasma-DENC comparisons, where samples and their corresponding DENCs were co-extracted across different days. For the plasma vs. plasma-DENC comparison, tests that adjusted for DEB or ‘sequencing run’ instead of ‘DNA extraction day’ and that also included the interaction of these factors with ‘sample type’, were also performed.

### Identification of high-confidence plasma ASVs

To search for ASVs that were more likely to be genuinely present in plasma instead of having originated from contaminant sources, we applied three independent bioinformatics filters to each one of the ASVs observed across plasma samples. These criteria have been applied for the study of other low-biomass microbiomes [18, 26] and have been recommended in this context, as strategies for addressing the issue of contamination [16, 21, 32].

Criterion (i) consisted of the identification of ASVs with no significant differential abundance associated with any technical variables that cause batch effects (i.e. sequencing run, DEB and ‘DNA extraction day’). For this, the limma R package (version 3.40.2) [23] was used. Library sizes were normalised using the trimmed mean of log expression ratios (TMM) method [48]. ASV counts were transformed to log2-counts per million (CPM) with associated precision weights using voom [49]. To account for the high sparsity of the ASV-by-samples counts that can underestimate the ‘genewise’ (in this case the ‘ASV-wise’) variances, a correction was applied [50]. To account for repeated measurements (i.e. different samples belonging to the same patient), the limma duplicateCorrelation function in which the ‘patient IDs’ were blocks was used to obtain a consensus correlation which was incorporated to the lmFit function and empirical Bayes moderated t statistics. Because we had an experimental design in which ‘DNA extraction day’ was nested in DEB and this was in turn, nested in ‘sequencing run’, a single linear regression model with ‘DNA extraction day’ as the only factor was built and the comparisons of interest were defined as contrasts. Whilst for testing the effect of DEB, contrasts corresponding to all possible DEB combinations were performed; for testing the effect of ‘DNA extraction day’, contrasts were only made for days within DEBs. *P* values were adjusted with the Benjamini and Hochberg method to control the FDR. An ASV with an FDR ≤ 0.05 in any of the contrasts made to test the effect of each one of the three technical variables (i.e. ‘DNA extraction day’, DEB and ‘sequencing run’) was considered as a batch effect.

Criterion (ii) consisted of the identification of ASVs with a higher prevalence in plasma vs. plasma-DENC samples, and for this, the isContaminant function (with method = ‘prevalence’, threshold = 0.55 and batch = DEB) of the decontam R package (version 1.4.0) [22] was used. To each ASV, this function applies a chi-square statistic (or a Fisher’s exact test in case of low number of samples) on the 2 × 2 presence-absence table in true samples and negative controls in which a score statistic *P* is defined as the tail probability of the chi-square distribution at that value. Using the ‘batch’ parameter of the isContaminant function, a score statistic was computed for each DEB independently and then combined by taking the minimum score across batches.

Criterion (iii) consisted of the identification of ASVs that across patient-matching DEBs (batches A and B or F and G) showed agreement of detection between replicates. For this, we used the kappa2 function of the irr R package (version 0.84.1) which implements the Cohen’s kappa coefficient which is an index of interrater agreement between two raters on categorical data. ASVs with a kappa score > 0.4 and a *p* values < 0.05 were considered to meet the criterion.

ASVs that met criterion (i) and (ii), and for ASVs present only in patient-matching DEBs A and B or F and G that also met criterion (iii), were selected. These ASVs were then taxonomically classified using an updated version of the Silva database (138 SSU Ref NR 99 515F/806R release) using both a Naive Bayes classifier (qiime feature-classifier classify-sklearn) and a consensus BLAST approach (qiime feature-classifier classify-consensus-blast with parameters --p-perc-identity 0.9 --p-min-consensus 0.8). Using a manually built consensus of the classifications obtained with these two approaches (see Additional file 2: Table S3 and Additional file 3: Table S7), the filtered ASVs were then subjected to criterion (iv) which consisted of the classification of each ASV in one of six mutually exclusive categories. These categories were defined based on the presence of the ASV’s taxonomic classification in a previously published list of ‘common contaminant taxa’ in low-biomass microbiome studies [16] and in a literature search for evidence that the taxa can be a ‘human pathogen or commensal’. This literature search only included high-biomass microbiome studies (e.g. intestinal tract, oral cavity) rather than studies that analysed blood or plasma samples. The definition of these categories is stated in Additional file 2: Table S3. ASVs in the ‘likely contaminant’ or ‘no evidence of commensal or pathogen’ were discarded and all the remaining ASVs were considered as high-confidence plasma ASVs.

### Measurement of microbial DNA concentration

FAM-labelled fluorophore reporter droplet digital PCR (ddPCR) assays to validate the 16S-rRNA gene sequencing results of high-confidence plasma ASVs classified as a *Faecalibacterium* (DADA2 feature ID: 59777186ad2e0947e97615b5d6225136) and *Clostridium* sensu stricto 9 (DADA2 feature ID: 82a260faafef55efd1b8176ecb781ecf) were custom designed. For this, a multiple sequence alignment and a phylogenetic tree built from the representative sequences of the ASVs obtained from plasma, plasma-DENC and NTC samples were obtained using ‘qiime alignment mafft’ and ‘qiime fragment-insertion sepp’ with the Greengenes 13_8 99% reference phylogeny, respectively. Based on the phylogenetic tree, the aligned sequences of the clade where the high-confidence plasma ASV was located and neighbouring clades were selected. This subset of the multiple sequence alignment was then manually inspected for primer/probes target regions that would maximise the number of mismatches between the high-confidence plasma ASV and neighbour ASVs. To control for the specificity of the assay, positive and negative gBlock gene fragments (Integrated DNA Technologies) were synthetized using the DNA sequence of the high-confidence plasma ASV and that of a neighbouring-clade ASV, respectively. For the *Faecalibacterium* (DADA2 feature ID: 59777186ad2e0947e97615b5d6225136) assay, Forward, Reverse and Probe sequences for this ASV-specific ddPCR assay were ACTGGGTGTAAAGGGAGCGC, GAATTCCGCCTACCTCTGCAC and AAGACAAGTTGGAAGTGAAATCCATGGGC, respectively. For the *Clostridium* sensu stricto 9 (DADA2 feature ID: 82a260faafef55efd1b8176ecb781ecf) assay, Forward, Reverse and Probe sequences for this ASV-specific ddPCR assay were AGCTTAACTTGGGTGCTGCATTTG, CTGTTTGCTCCCCACGCTTTCAT and TTCCACTTACCTCTCCTGCACTCTAGATAT, respectively. The assay was multiplexed with a HEX-labelled fluorophore reporter ddPCR assay designed using primers and a probe from a previously published universal 16SrRNA TaqMan quantitative PCR assay [51]. This assay was also used to measure the absolute levels of microbial DNA across sample types and DENCs.

ddPCR analysis was performed using the Bio-Rad Droplet Digital PCR system following manufacturer’s protocols. ddPCR reactions were 25 μL aqueous volumes that contained final concentrations of 1x ddPCR supermix for probes (without dUTP) (Bio-Rad), 0.9 μM each primer and 0.25 μM probe. The thermal cycling conditions were 95 °C: for 10 min, followed by 40 cycles of 95 °C for 15 seconds and annealing for 1 min at 65 °C. Each sample was analysed by at least two technical replicates comprising of at least 10000 individual reactions. A Poisson correction was applied to determine the number of amplifiable molecules, which was used to further derive the number of copies of DNA carrying a particular ASV per millilitre of plasma. Data analysis was carried out using the QuantaSoft Software, version 1.7 (Bio-Rad). An ASV was defined as detectable if there was ≥ 2 copies detected across the duplicate reactions. To test for differences in the concentration of gene copies between plasma and plasma-DENC, GEEs were applied by employing the same function and regression model used to test differences in alpha diversity.

## Supplementary Information

**Additional file 1: Figure S1.** 16S rRNA gene sequencing of an *E. coli* genomic DNA dilution series. **Figure S2.** Number of 16S rRNA gene quality-filtered reads obtained across sample types. **Figure S3.** Taxonomic profile of a 20 strain evenly mixed mock community sequenced alongside study samples. **Figure S4.** Taxonomic profiles of patient-matching plasma, stool and saliva samples and respective DENCs. **Figure S5.** Batch effects due to DNA extraction day for plasma samples based on Bray-Curtis dissimilarity analysis. **Figure S6.** Taxonomic profile of plasma samples and their corresponding DENCs from DEBs C-E. **Figure S7.** Batch effects due to sequencing run and DEB based on Aitchison distance analysis. **Figure S8.** Prevalence of ASVs in plasma vs. DENC for DEB A to D. **Figure S9.** Sequencing and in silico decontamination results from the extension cohort of healthy individuals and melanoma patients. **Table S1.** Clinical characteristics of melanoma patients from the initial study cohort. **Table S2.** Plasma ASVs and reads shared with stool and saliva samples per patient. **Table S4.** Clinical characteristics of the melanoma patients from the extension cohort. **Table S5.** *P*-values resulting from hypothesis tests on alpha and beta diversity estimates obtained from the extension cohort. **Table S6.** Number of plasma ASVs from the extension cohort meeting each decontamination criterion separately and in combination.

**Additional file 2: Table S3.** Decontamination criteria outcome for ASVs that passed the in silico decontamination strategy applied to the plasma samples of the initial cohort.

**Additional file 3: Table S7.** Decontamination criteria outcome for ASVs that passed the in silico decontamination strategy applied to plasma samples of the extension cohort.

**Additional file 4.** Review history.

## Data Availability

All sequencing data generated in this study are available at the National Center for Biotechnology Information Sequence Read Archive (https://www.ncbi.nlm.nih.gov/sra) under bioproject number PRJNA666045 [52]. The data and source R code for reproducing the main results are available at https://github.com/ezozayav/Detection_of_cfmDNA [53].
