# Supplementary material for: Detection of cell-free microbial DNA using a contaminant-controlled analysis framework
Source: Genome Biol. 2021 Jun 23;22:187. doi: 10.1186/s13059-021-02401-3 (PMC8220693; doi:10.1186/s13059-021-02401-3)
Supplement: Supplementary file 1 — Additional file 1: Figure S1. 16S rRNA gene sequencing of an E. coli genomic DNA dilution series. Figure S2. Number of 16S rRNA gene quality-filtered reads obtained across sample types. Figure S3. Taxonomic profile of a 20 strain evenly mixed mock community sequenced alongside study samples. Figure S4. Taxonomic profiles of patient-matching plasma, stool and saliva samples and respective DENCs. Figure S5. Batch effects due to DNA extraction day for plasma samples based on Bray-Curtis dissimilarity analysis. Figure S6. Taxonomic profile of plasma samples and their corresponding DENCs from DEBs C-E. Figure S7. Batch effects due to sequencing run and DEB based on Aitchison distance analysis. Figure S8. Prevalence of ASVs in plasma vs. DENC for DEB A to D. Figure S9. Sequencing and in silico decontamination results from the extension cohort of healthy individuals and melanoma patients. Table S1. Clinical characteristics of melanoma patients from the initial study cohort. Table S2. Plasma ASVs and reads shared with stool and saliva samples per patient. Table S4. Clinical characteristics of the melanoma patients from the extension cohort. Table S5. P-values resulting from hypothesis tests on alpha and beta diversity estimates obtained from the extension cohort. Table S6. Number of plasma ASVs from the extension cohort meeting each decontamination criterion separately and in combination. [file 13059_2021_2401_MOESM1_ESM.docx]

**Additional file 1**

**Table of contents**

**Figure S1**. 16S rRNA gene sequencing of an *E. coli* genomic DNA dilution series.

**Figure S2**. Number of 16S rRNA gene quality-filtered reads obtained across sample types.

**Figure S3.** Taxonomic profile of a 20 strain evenly mixed mock community, sequenced alongside study samples.

**Figure S4.** Taxonomic profiles of patient-matching plasma, stool and saliva samples and respective DENCs.

**Figure S5.** Batch effects due to DNA extraction day for plasma samples based on Bray-Curtis dissimilarity analysis.

**Figure S6.** Taxonomic profile of plasma samples and their corresponding DENCs from DEBs C-E.

**Figure S7.** Batch effects due to sequencing run and DEB based on Aitchison distance analysis.

**Figure S8.** Prevalence of ASVs in plasma vs DENC for DEB A to D.

**­­Figure S9.** Sequencing and *in silico* decontamination results from the extension cohort of healthy individuals and melanoma patients.

**Table S1.** Clinical characteristics of melanoma patients from the initial study cohort.

**Table S2.** Plasma ASVs and reads shared with stool and saliva samples per patient.

**Table S4.** Clinical characteristics of melanoma patients from the extension cohort.

**Table S5.** P-values resulting from hypothesis tests on alpha and beta diversity estimates obtained from the extension cohort.

**Table S6.** Number of plasma ASVs form the extension cohort that met each decontamination criterion separately and in combination.

**Figure S1**. **16S rRNA gene sequencing of an** ***E. coli* genomic DNA dilution series.** On a square-root transformed scale, the y-axis shows the number of quality-filtered 16S rRNA gene reads obtained from a ten-fold dilution series of *E. coli* genomic DNA. The genus-level taxonomic classification of all ASVs obtained is shown which, apart from those classified as *E. coli*, are assumed to represent contaminants. ASVs with an abundance across samples below 0.01% were removed.

**Figure S2. Number of 16S rRNA gene quality-filtered reads obtained across sample types.** DNA Extraction Negative Controls (DENCs) consisted of DNA extractions (using the kit corresponding to each sample-type) where nuclease-free water was the only input used. Non-Template Controls (NTCs) were sequencing PCR reactions where nuclease-free water was the only input used. The dot inside the boxes represents the mean.

**Figure S3. Taxonomic profile of a 20 strain evenly mixed mock community sequenced alongside study samples.** **A)** Abundance (i.e. number of reads) of all ASVs recovered across mock-community sequencing replicates. On the x-axis, the taxonomic classification of each ASV is shown. Excepting the genus *Propionibacterium*, which was not detected, the taxonomic classification of the recovered ASVs corresponded to all strains stated in the mock community’s product information sheet. Two and one extra ASVs were recovered for genera *Bacteroides* and *Clostridium*, respectively. The ASV classified as a *Flavobacterium* was not stated in the mock community’s product information sheet and therefore was expected to represent a contaminant. **B)** Abundance (i.e. number of reads) distribution of the 20 most abundant ASVs in each one of the sequencing replicates included in the study. Boxes within each vertical bar represent ASVs’ abundances. Within vertical bars, boxes with the same colour, represent different ASVs that were classified to the same genus (e.g. *Bacteroides*). ASVs with an abundance across replicates below 0.01% were removed and replicates were rarefied to a minimum sample size of 9,593 reads.

**Figure S4. Taxonomic profiles of patient-matching plasma, stool and saliva samples and respective DENCs.** The taxonomic composition based on 16S rRNA gene sequencing of samples are shown at the levels of **A)** Class**, B)** Family and **C)** Genus. For plasma, only batches A and B are shown. The top 15 most abundant taxa are shown for Class and the top 20 most abundant taxa are shown for Family and Genus.

**Figure S5. Batch effects due to DNA extraction day for plasma samples based on Bray-Curtis dissimilarity analysis.** Shown is a dendrogram based on hierarchical clustering analysis (UPGMA) of pairwise Bray-Curtis dissimilarities for all plasma samples and their corresponding DENCs at the ASV-level highlighting batch effects due to DNA extraction day.

**Figure S6. Taxonomic profile of plasma samples and their corresponding DENCs from DEBs C-E.** Taxonomic profiles based on 16S rRNA gene sequencing are shown at the level of **A)** Phylum**, B)** Class**, C)** Family and **D)** Genus. The top 15 most abundant taxa are shown for Phylum and Class and the top 20 most abundant taxa are shown for Family and Genus.

**Figure S7.** **Batch effects due to sequencing run and DEB based on Aitchison distance analysis.** Dendrogram based on hierarchical clustering analysis (UPGMA) of Aitchison distances for all plasma samples (DEBs A-E) and their corresponding DENCs at the ASV-level highlighting batch effects due to sequencing run and DNA extraction batch (DEB).

**Figure S8. Prevalence of ASVs in plasma vs DENC for DEB A to D.** Plots show the prevalence across different DENC replicates (x-axis) and plasma samples (y-axis) for all ASVs found in DEBs A to D (corresponding to panels A to D). ASVs classified as “real” or “contaminant” by decontam’s prevalence method are depicted in red and blue, respectively. The abundance which is represented by the size of the data points, is the average relative abundance (i.e. number of reads normalized by the sample size) of an ASV across plasma samples.


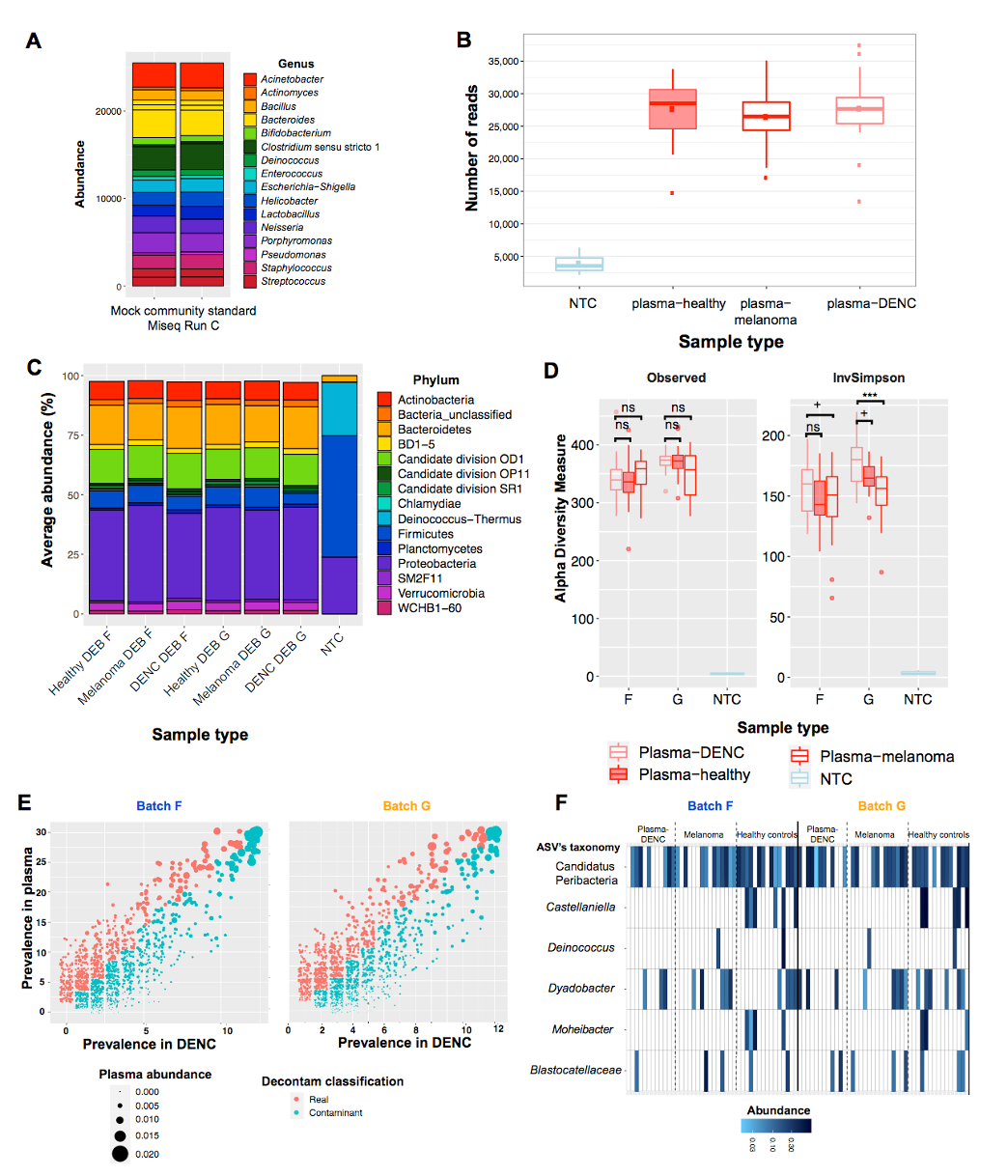


**­Figure S9. Sequencing and *in silico* decontamination results from the extension cohort of melanoma and healthy controls. A)** Taxonomic profile of a 20 strain evenly mixed mock community sequenced alongside study samples from the extension cohort. Abundance (i.e. number of reads) distribution of the 20 most abundant ASVs in each one of the sequencing replicates included in the study are shown. Boxes within each vertical bar represent ASV abundances. Within vertical bars, boxes with the same colour, represent different ASVs that were classified to the same genus (e.g. *Bacteroides*). **B)** Number of 16S rRNA gene quality-filtered reads obtained from samples sequenced. DNA Extraction Negative Controls (DENCs) consisted of DNA extractions where nuclease-free water was the only input used. Non-Template Controls (NTCs) were sequencing PCR reactions where nuclease-free water was the only input used. The dot inside the boxes represents the mean. **C)** Taxonomic profiles of plasma, respective DENCs and non-template control (NTC) in the extension cohort at the levels of Phylum. **D)** Alpha diversity measurements of all plasma samples and their corresponding DENCs across the two DNA extraction batches F and G based on the number of observed ASVs (richness) and inverse Simpson’s Index (diversity). “+” p between 0.1-0.05, *** p<0.001, ns - not significant. **E)** Prevalence of ASVs in plasma vs DENC for DEBs F and G. Plots show the prevalence across different DENC replicates (x-axis) and plasma samples (y-axis) for all ASVs found in DEBs F and G. ASVs classified as “real” or “contaminant” by decontam’s prevalence method are depicted in red and blue, respectively. The abundance which is represented by the size of the data points, is the average relative abundance (i.e. number of reads normalized by the sample size) of an ASV across plasma samples. **F)** The prevalence and abundance across plasma samples and DENCs of 6 plasma ASVs identified from the extension cohort using filtering criteria (i) to (iii) as described in the methods. The abundance represents a log10 transformation of the percentage of reads per plasma sample.

**Table S1. Clinical characteristics of melanoma patients from the initial study cohort.**

| **Melanoma Biomarker_ID** | **Gender** | **Age (at diagnosis of metastatic disease)** |  | **Melanoma biomarker_ID** | **Gender** | **Age (at diagnosis of metastatic disease)** | |
| --- | --- | --- | --- | --- | --- | --- | --- |
| 46 | M | 39 |  | 181 | M | | 75 |
| 63 | F | 65 |  | 182 | F | | 55 |
| 71 | M | 62 |  | 183 | M | | 53 |
| 79 | M | 53 |  | 186 | M | | 70 |
| 80 | M | 68 |  | 188 | F | | 34 |
| 81 | M | 62 |  | 194 | M | | 45 |
| 84 | M | 70 |  | 195 | M | | 55 |
| 90 | M | 59 |  | 198 | F | | 70 |
| 96 | M | 73 |  | 200 | M | | 70 |
| 98 | F | 50 |  | 202 | M | | 67 |
| 99 | F | 46 |  | 203 | F | | 66 |
| 108 | M | 81 |  | 204 | F | | 67 |
| 113 | M | 77 |  | 208 | M | | 76 |
| 114 | M | 59 |  | 209 | M | | 60 |
| 121 | M | 70 |  | 210 | M | | 53 |
| 122 | M | 80 |  | 212 | M | | 38 |
| 123 | F | 51 |  | 213 | M | | 70 |
| 125 | F | 78 |  | 215 | M | | 57 |
| 126 | M | 34 |  | 218 | M | | 49 |
| 133 | M | 62 |  | 222 | M | | 50 |
| 134 | M | 55 |  | 225 | F | | 59 |
| 136 | M | 73 |  | 234 | M | | 47 |
| 140 | M | 57 |  | 238 | M | | 76 |
| 143 | F | 34 |  | 239 | M | | 48 |
| 144 | M | 61 |  | 242 | M | | 76 |
| 145 | M | 71 |  | 244 | M | | 61 |
| 147 | F | 75 |  |  |  | |  |
| 149 | M | 40 |  |  |  | |  |
| 151 | M | 59 |  |  |  | |  |
| 152 | M | 29 |  |  |  | |  |
| 154 | F | 69 |  |  |  | |  |
| 155 | M | 68 |  |  |  | |  |
| 157 | M | 71 |  |  |  | |  |
| 160 | F | 72 |  |  |  | |  |
| 161 | F | 72 |  |  |  | |  |
| 163 | M | 55 |  |  |  | |  |
| 164 | M | 69 |  |  |  | |  |
| 165 | M | 46 |  |  |  | |  |
| 166 | M | 75 |  |  |  | |  |
| 170 | M | 72 |  |  |  | |  |
| 175 | F | 62 |  |  |  | |  |
| 176 | M | 71 |  |  |  | |  |
| 180 | M | 70 |  |  |  | |  |

**Table S2. Plasma ASVs and reads shared with patient-matching stool and saliva samples per patient.**

| **Plasma sample** | **ASVs shared with stool (%)** | **% of reads shared with stool** | **ASVs shared with saliva (%)** | **% of reads shared with saliva** |
| --- | --- | --- | --- | --- |
| 90_1 | 13 (10.7) | 9.9 | 1 (0.8) | 0.1 |
| 90_2 | 2.5 (1.5) | 1.35 | 0.5 (0.8) | 0.2 |
| 200_1 | 15 (11.5) | 15.5 | 0 (0) | 0 |
| 202_1 | 6.5 (7.05) | 18.3 | 1.5 (1.85) | 0.7 |
| 204_1 | 4 (5.5) | 4.5 | 0 (0) | 0 |
| 210_1 | 0.5 (1.45) | 0.05 | 0 (0) | 0 |
| 213_1 | 1.5 (1.75) | 2.3 | 0 (0) | 0 |
| 215_1 | 5 (4.55) | 16.65 | 3.5 (2.5) | 9.7 |
| 218_1 | 5 (3.35) | 0.85 | 4.5 (3.4) | 2.3 |
| 222_1 | 1 (1) | 0.45 | 0.5 (0.75) | 0.25 |
| 225_1 | 9 (4.65) | 22.45 | 0.5 (0.15) | 13.7 |
| 234_1 | 2 (1.55) | 0.55 | 1 (0.35) | 13.75 |
| 238_1 | 0.5 (0.2) | 0 | 1.5 (1.25) | 24.25 |
| 239_1 | 1.5 (1.3) | 16.65 | 1 (1.15) | 0.1 |
| 242_1 | 4.5 (3.55) | 3.7 | 2.5 (2.7) | 0.65 |
| 244_1 | 0 (0) | 0 | 1 (0.8) | 14.6 |

For each patient, the average number of ASVs shared between sample types across the patient-matching plasma samples from DEBs A and B was calculated.

**Table S4. Clinical characteristics of melanoma patients from the extension cohort.**

| **Melanoma Biomarker_ID** | **Gender** | **Age (at diagnosis of metastatic disease)** |
| --- | --- | --- |
| 139 | M | 60 |
| 37 | M | 76 |
| 103 | F | 62 |
| 159 | M | 75 |
| 109 | F | 58 |
| 137 | M | 59 |
| 128 | F | 68 |
| 138 | M | 66 |
| 49 | F | 71 |
| 79 | M | 53 |
| 117 | F | 45 |
| 50 | F | 40 |
| 75 | M | 57 |
| 94 | M | 50 |
| 60 | M | 64 |

**Table S5. P-values resulting from hypothesis tests on alpha and beta diversity estimates obtained from the extension cohort.**

|  | **DEB** | **Ext-run** | **Plasma vs Plasma-DENC** | | **Healthy vs DENC** | | **Melanoma vs DENC** | | **Healthy vs melanoma** | |
| --- | --- | --- | --- | --- | --- | --- | --- | --- | --- | --- |
| Adj by | pl vs DENC | pl vs DENC | DEB | ext-id | DEB | ext-id | DEB | ext-id | DEB | ext-id |
| Observed | **0.019** | **<0.001** | 0.733 | 0.708 | 0.939 | 0.929 | 0.586 | 0.534 | 0.687 | 0.625 |
| Inv-Simpson | **0.010** | **<0.001** | **0.004** | **0.002** | **0.035** | **0.019** | **0.008** | **0.001** | 0.095 | **0.042** |
| Bray Curtis | 0.052 | **0.005** | **0.001** | **0.001** | **0.026** | **0.014** | **0.003** | **0.002** | 0.397 | 0.357 |
| Aitchison | 0.165 | 0.247 | **0.001** | **0.001** | **0.001** | **0.001** | **0.002** | **0.001** | 0.132 | 0.134 |

Significance tests on the number of observed ASVs and on the inverse of the Simpson diversity index were performed using Generalized Estimating Equations (GEE) with the geepack R package. Bray Curtis and Aitchison significance tests were performed using a Repeated Measurement Aware PERMANOVA test implemented in R.

**Table S6. Number of plasma ASVs form the extension cohort that met each decontamination criterion separately and in combination.**

|  | **All ASVs (%)** | **Low abundance ASVs (%)** | **Med. abundance ASVs (%)** | **High abundance ASVs (%)** |
| --- | --- | --- | --- | --- |
| Total | 1675 (100) | 1491 (100) | 178 (100) | 6 (100) |
| **Criterion (i)** | | | | |
| No batch-effect by DEB | 1675 (100) | 1491 (100) | 178 (100) | 6 (100) |
| No batch-effect by DNA ext. run | 1674 (99.94) | 1490 (99.93) | 178 (100) | 6 (100) |
| No batch-effect by any tech. var. | 1674 (99.94) | 1490 (99.93) | 178 (100) | 6 (100) |
| **Criterion (ii)** | | | | |
| Decontam in DEB F | 676 (40.36) | 585 (39.24) | 91 (51.12) | 0 (0) |
| Decontam in DEB G | 693 (41.37) | 598 (40.11) | 93 (52.25) | 2 (33.33) |
| Decontam across DEBs | 347 (20.72) | 300 (20.12) | 47 (26.4) | 0 (0) |
| **Criterion (iii)** | | | | |
| Sample association | 38 (2.27) | 34 (2.28) | 4 (2.25) | 0 (0) |
| Complete bioinformatics decontamination strategy | 6 (0.36) | 5 (0.34) | 1 (0.56) | 0 (0) |
| Final list after literature-based filter | 2 (0.12) | 1 (0.07) | 1 (0.56) | 0 (0) |

low abundance: < 0.1%, med abundance: 0.1% - 1%, high abundance: > 1%, DNA ext. run: DNA extraction run, tech. var.: technical variable.
