# Supplementary material for: Detection of cell-free microbial DNA using a contaminant-controlled analysis framework
Source: Genome Biol. 2021 Jun 23;22:187. doi: 10.1186/s13059-021-02401-3 (PMC8220693; doi:10.1186/s13059-021-02401-3)
Supplement: Supplementary file 4 — Additional file 4. Review history. [file 13059_2021_2401_MOESM4_ESM.docx]

Review History

**First round of review**

**Reviewer 1**

**Are you able to assess all statistics in the manuscript, including the appropriateness of statistical tests used?**

Yes, and I have assessed the statistics in my report.

**Comments to author:**

The methods of this paper are well described with the necessary controls included and interpreted accurately. The conclusions are supported by the data, and there are sufficient details to repeat the experiments. The contents will be of high interest to others in this field, while highlighting the need for better reagents and improved collection and processing controls to investigate the exciting area of microbial biomarkers and signals in low-biomass communities, in particular in blood.

Overall the authors are to be commended on this effort and their excellent use of controls, paired-samples, appropriate statistics and clear language. There are only a few, relatively minor comments I'd like to see addressed.

1. The samples in this study are all from individuals with late stage cancer. As such, the abundance and composition of the microbes present may not be reflective of those to be found in healthy individuals. This limitation should be noted since samples from individuals with a lower abundance of bacteria may be below the floor established by the heavy background contamination seen in the reagents.

2. The omission of Cutibacterium (nee Propionibacterium) using V4 primers has been noted elsewhere, so a small reference to this issue may alleviate any concerns on missing that genus in the mock community samples (Meisel et al 2017 J Invest Derm PMID: 26829039)

3. Lines 304-309: While the authors correctly note the benefits of using 16S sequencing vs shotgun approaches used in the prior reports, it should be noted that 16S sequencing will result in limiting the microbial investigation to only bacteria whose 16S V4 subfragment is available for isolation in the blood, either from intact or lysed/damaged microbes. The works in refs 12 and 13 demonstrate that the microbial diversity seen with only 16S sequencing may not accurately represent the range of microbial DNA that may be detected in circulation, but is a more affordable option for studies focused only on the investigation of microbial signatures from relatively intact genomes.

**Reviewer 2**

**Are you able to assess all statistics in the manuscript, including the appropriateness of statistical tests used?**

Yes, and I have assessed the statistics in my report.

**Comments to author:**

In this study, the authors performed a pilot analysis of whether DNA from the microbiome can be detected via 16S rRNA sequencing of cell-free DNA. Low-biomass microbiome sequencing studies have a large number of methodological challenges, which the authors addressed through the use of controls and stringent analysis. The filtering criteria for identifying high-confidence plasma ASVs are logical and well motivated. The description of the the extensive contaminants and batch effects is valuable for the field and worth reporting. There are several concerns to resolve prior to publication:

There are some issues with the framing and conclusions of the study. The title is somewhat misleading - it is not clear that the authors have demonstrated "Accurate" detection of cell-free microbial DNA, because there was no positive control or gold standard to validate their approach (including the in silico workflow), other than the ddPCR for a single ASV.

The framing around cancer biomarkers is confusing, since the specific findings are not interpretable without comparison with healthy controls. What is the hypothesis about the nature of the biomarkers that it may be possible to detect with this method? A biomarker would typically consistent of differences in abundance and/or presence/absence in a particular biological feature, and the authors have not demonstrated their method can accurate distinguish either of these properties with their method.

The matched stool and saliva samples are a missed opportunity to validate the findings in plasma samples. Do plasma samples in Batches A and B share more ASVs with their patient-matched stool and saliva than with other samples of those types? Were ASVs in those clades more likely to be identified as high confidence by other metrics?

Relatedly, the taxonomic analysis does not take advantage of sequence-level information. In addition to a literature search for whether a particular species has been identified as a commensal or pathogen, it would be useful to use a sequence-based search such as redbiom or even NCBI BLAST for the ASV sequences in question, since many well-documented human gut ASVs still have ambiguous or shifting taxonomic designations.

In low-biomass samples with very low read numbers and large amounts of contaminants, compositionality can have a strong effect. It would be helpful to compare the Bray-Curtis results, especially the clustering of plasma samples and negative controls, with an ordination method using Aitchison log-ratio distances (CLR or PhILR).

Minor comments:

How were the numbers of PCR cycles prior to sequencing chosen? Was this validated at all for these sample types?

How was the RMA-Aware PERMANOVA method implemented? How did it account for repeated measures?

How was the FDR cutoff chosen for the limma differential abundance analyses? It seems like an effect size threshold may be a better choice for this purpose since the key goal is to filter possibly batch-linked ASVs, regardless of the number of samples, batches and ASVs (which would all have varying effects on the p-values and FDR).

How was the Faecalibacterium ASV selected for ddPCR validation? The discussion should mention any factors that might influence whether this result is likely to be generalizable to the other identified ASVs.

Were the high-confidence ASVs identified here similar in taxonomy or other properties to the taxa identified by Poore et al in their metagenomic study?

**Authors Response**

**Point-by-point responses to the reviewers’ comments:**

Reviewer #1:

The methods of this paper are well described with the necessary controls included and interpreted accurately. The conclusions are supported by the data, and there are sufficient details to repeat the experiments. The contents will be of high interest to others in this field, while highlighting the need for better reagents and improved collection and processing controls to investigate the exciting area of microbial biomarkers and signals in low-biomass communities, in particular in blood.

Overall the authors are to be commended on this effort and their excellent use of controls, paired-samples, appropriate statistics and clear language. There are only a few, relatively minor comments I'd like to see addressed.

*We thank the reviewer for their positive comments and for highlighting the high interest of our findings to others in this field. We have fully addressed the comments raised as detailed below.*

1. The samples in this study are all from individuals with late stage cancer. As such, the abundance and composition of the microbes present may not be reflective of those to be found in healthy individuals. This limitation should be noted since samples from individuals with a lower abundance of bacteria may be below the floor established by the heavy background contamination seen in the reagents.

*We agree with the reviewer that the abundance and composition of microbial DNA in plasma samples from those with late stage could be different to that of healthy individuals. As such, we have now performed additional analyses of samples from healthy individuals (n=15). To allow assessment of batch effects due to sample processing and DNA extraction, the samples from healthy individuals were analysed in parallel to a new cohort of samples from patients with melanoma (n=15) which were processed at the same time, so that batch effects could be accurately assessed and our contaminant-controlled analysis framework could be applied. This then allowed us to directly compare the representation of microbial DNA between both groups (healthy vs melanoma) in this cohort.*

*As expected, both plasma samples from healthy individuals and melanoma patients were highly susceptible to contaminant DNA. Importantly, in both plasma samples from healthy individuals and melanoma patients, we were again able to identify a small but significant difference between the microbiome composition of cell free microbial DNA (cfmDNA) and DNA extraction negative controls (DENCs). After filtering for contaminant ASVs through our analytical and informatic framework, a total of 347 ASVs passed criteria (i) and (ii), a total of 6 high-confidence ASVs passed criteria (i), (ii) and (iii) (Fig. S9F) and a total of 2 high-confidence ASVs passed all four criteria (Additional file 1: Table S6 and Additional file 3: Table S7).*

*To identify differentially abundant ASVs between healthy and melanoma samples, we applied limma hypothesis testing on the set of 347 ASVs that met criteria (i) and (ii). Using limma linear models that separately adjusted for DNA-extraction batch and DNA-extraction-run, one differentially abundant ASV was found after correcting for multiple testing. This high confidence ASV was only present in healthy controls and was absent from any of the plasma DENC samples. It passed our three main bioinformatic decontamination criteria and was classified into the Castellaniella genus.*

*In summary, our additional data shows that our analytic approach can detect high confidence ASVs in plasma samples from both healthy individuals and late stage cancer patients. Whilst we have now shown the feasibility of our approach in both samples from healthy individuals and cancer patients, additional analyses with larger cohorts will be needed to identify and validate distinct ASVs associated with cancer vs healthy individuals.*

2. The omission of Cutibacterium (nee Propionibacterium) using V4 primers has been noted elsewhere, so a small reference to this issue may alleviate any concerns on missing that genus in the mock community samples (Meisel et al 2017 J Invest Derm PMID: 26829039)

*We thank the reviewer for this reference. We have now cited this reference in relation to our findings with the mock community sample (lines 145-151).*

3. Lines 304-309: While the authors correctly note the benefits of using 16S sequencing vs shotgun approaches used in the prior reports, it should be noted that 16S sequencing will result in limiting the microbial investigation to only bacteria whose 16S V4 subfragment is available for isolation in the blood, either from intact or lysed/damaged microbes. The works in refs 12 and 13 demonstrate that the microbial diversity seen with only 16S sequencing may not accurately represent the range of microbial DNA that may be detected in circulation, but is a more affordable option for studies focused only on the investigation of microbial signatures from relatively intact genomes.

*We agree with the reviewer’s comment and have provided additional detail in the discussion to highlight this important limitation of 16S sequencing (lines 441-443).*

Reviewer #2:

In this study, the authors performed a pilot analysis of whether DNA from the microbiome can be detected via 16S rRNA sequencing of cell-free DNA. Low-biomass microbiome sequencing studies have a large number of methodological challenges, which the authors addressed through the use of controls and stringent analysis. The filtering criteria for identifying high-confidence plasma ASVs are logical and well motivated. The description of the the extensive contaminants and batch effects is valuable for the field and worth reporting.

*We thank the reviewer for highlighting the strengths of our stringent analysis approach given the challenges of low-biomass microbiome sequencing, and the value of our findings for the field.*

There are several concerns to resolve prior to publication:

1. There are some issues with the framing and conclusions of the study. The title is somewhat misleading - it is not clear that the authors have demonstrated "Accurate" detection of cell-free microbial DNA, because there was no positive control or gold standard to validate their approach (including the in silico workflow), other than the ddPCR for a single ASV.

*This research area of the study of microbial biomarkers from low-biomass samples, in particular blood, is an emerging field. To our knowledge, there are no gold standards and no positive controls of cell free microbial DNA that can currently be used to validate our approach. To address the reviewer’s concern we have modified the title of the manuscript. In addition, we have performed additional experiments and analyses to provide orthogonal validation of our findings. This has included the validation of another ASV through the use of ddPCR (Clostridium sensu stricto 9)(updated Figure 4F). Furthermore, we have sequenced a dilution series of E-coli DNA to demonstrate that our sequencing approach has the required sensitivity to detect bacterial reads down to a similar level found in our plasma DNA samples via ddPCR, i.e. ~10-1 copies/ul of DNA (Additional File 1, Figure S1).*

2. The framing around cancer biomarkers is confusing, since the specific findings are not interpretable without comparison with healthy controls. What is the hypothesis about the nature of the biomarkers that it may be possible to detect with this method? A biomarker would typically consistent of differences in abundance and/or presence/absence in a particular biological feature, and the authors have not demonstrated their method can accurate distinguish either of these properties with their method.

*The primary aim of our analysis was to establish a novel contaminant-controlled analysis framework to examine the existence of plasma derived cell free microbial DNA and detect high confidence cfmDNA ASVs. Through the use of plasma samples from cancer patients, in parallel to stool and saliva samples from the same patients we have developed and refined this workflow.*

*As indicated by the reviewer, a logical extension of this analytical framework would be to apply it to the analysis of samples from cancer patients versus healthy individuals for the identification of cancer specific microbial biomarkers. Whilst this was not the primary aim of our analysis, we have now provided additional data to support this concept. Specifically, we have performed additional analyses of samples from healthy individuals (n=15). To allow assessment of batch effects due to sample processing and DNA extraction, the samples from healthy individuals were analysed in parallel to a new cohort of samples from patients with melanoma (n=15) which were processed at the same time, so that batch effects could be accurately assessed and our contaminant-controlled analysis framework could be applied. This then allowed us to directly compare the representation of microbial DNA between both groups (healthy vs melanoma) in this cohort.*

*As expected, both plasma samples from healthy individuals and melanoma patients were highly susceptible to contaminant DNA. Importantly, in both plasma samples from healthy individuals and melanoma patients, we were again able to identify a small but significant difference between the microbiome composition of cell free microbial DNA (cfmDNA) and DNA extraction negative controls (DENCs). After filtering for contaminant ASVs through our analytical and informatic framework, a total of 347 ASVs passed criteria (i) and (ii), a total of 6 high-confidence ASVs passed criteria (i), (ii) and (iii) (Fig. S9F) and a total of 2 high-confidence ASVs passed all four criteria (Additional file 1: Table S6 and Additional file 3: Table S7).*

*To identify differentially abundant ASVs between healthy and melanoma samples, we applied limma hypothesis testing on the set of 347 ASVs that met criteria (i) and (ii). Using limma linear models that separately adjusted for DNA-extraction batch and DNA-extraction-run, only one differentially abundant ASV was found after correcting for multiple testing. This high confidence ASV was only present in healthy controls and was absent from any of the plasma DENC samples. It passed our three main bioinformatic decontamination criteria and was classified into the Castellaniella genus.*

*In summary, whilst our additional data shows the feasibility of our approach to discover cancer microbial biomarkers from plasma, further analyses with larger cohorts will be needed to identify and validate distinct ASVs associated with cancer vs healthy individuals. We have expanded the conclusions within the manuscript to illustrate these findings.*

The matched stool and saliva samples are a missed opportunity to validate the findings in plasma samples. Do plasma samples in Batches A and B share more ASVs with their patient-matched stool and saliva than with other samples of those types? Were ASVs in those clades more likely to be identified as high confidence by other metrics?

*To address this query, we have calculated matrixes of the number of ASVs shared between all plasma vs. stool samples and plasma vs. saliva samples from the patients in batch A before and after decontamination (See reviewer only Figure 1). The majority of the ASVs found to be shared between samples-types were potential contaminants as they were removed following our stringent decontamination analysis. After decontamination, the list of ‘high-confidence ASVs’ was small (n=31), and plasma samples did not share more ASVs with their patient-matched stool and saliva samples (highlighted by the diagonal in reviewer only Figure 1) compared with other samples of those types across the cohort. The same analysis was performed with the samples in batch B and showed similar results. As a result we have elected not to include this data in the manuscript. Analyses of a larger cohort of matched plasma, stool and saliva will be needed to further address this question.*

3. Relatedly, the taxonomic analysis does not take advantage of sequence-level information. In addition to a literature search for whether a particular species has been identified as a commensal or pathogen, it would be useful to use a sequence-based search such as redbiom or even NCBI BLAST for the ASV sequences in question, since many well-documented human gut ASVs still have ambiguous or shifting taxonomic designations.

*We thank the reviewer for this helpful comment. To improve the accuracy and robustness of the taxonomic classifications of the ASVs that passed our bioinformatics decontamination strategy, we re-classified these ASVs using the most up-to-date Silva database (138 SSU Ref NR 99 515F/806R release) using both a Naïve Bayes classifier (as before) and a consensus BLAST approach as implemented in QIIME2. The classifications (at the genus or higher taxonomic levels) obtained by these two approaches matched the original list of 31 ASVs that passed our bioinformatics decontamination strategy in the initial cohort except for two ASVs (described in detail below). All the classifications of the ASVs that passed our bioinformatics decontamination strategy in the new extension cohort matched. This additional analysis supports the robustness of the initially chosen Naïve Bayes classifier approach. The classifications resulting from the two approaches as well as a manually-built consensus from these two approaches have now been included in Additional file 2: Table S3 and Additional file 3: Table S7. The methods for the additional analyses have been detailed in the ‘Identification of high confidence plasma ASVs’ sub-section of the methods.*

*After re-assessing our literature-based criterion (iv) based on the new taxonomic classifications, only the ASVs with a DADA2 ASV ID of 06f9a55c4328c1d270ef70f7b2bad23f and d4b47821b9c53e2423fcdf8b449a41c1 had their literature-based contamination likelihood changed from ‘unknown’ to ‘mixed evidence’ and from ‘no evidence of commensal or pathogen’ to ‘likely contaminant’, respectively. These are shown in Additional file 2: Table S3.*

4. In low-biomass samples with very low read numbers and large amounts of contaminants, compositionality can have a strong effect. It would be helpful to compare the Bray-Curtis results, especially the clustering of plasma samples and negative controls, with an ordination method using Aitchison log-ratio distances (CLR or PhILR).

*We thank the reviewer for suggesting analysis with this alternative distance metric. We have now applied Aitchison log-ratio distances to our dataset, and included the results in a new supplementary figure (Additional File 1: Figure S7). Here, the UPGMA dendogram based on Aitchison distances shows a clustering pattern similar to the Bray-Curtis analysis. Importantly, a RMA-PERMANOVA test based on Aitchison distances, showed the same result as with the Bray-Curtis analysis with a significant difference in the effect of all three technical variables (i.e. sequencing run, DNA extraction batch and DNA extraction day) in the community structure, as well as a significant difference between plasma and plasma-DENC after separately adjusting for each one of the technical variables. Overall, this new analysis further supports our findings that despite batch effects due to contamination, there is still a significant difference between the microbiome composition of plasma and negative controls.*

Minor comments:

5. How were the numbers of PCR cycles prior to sequencing chosen? Was this validated at all for these sample types?

*The PCR protocol used for sequencing was adapted from the 16S rRNA gene Illumina amplicon protocol of the Earth Microbiome Project and this is now reflected in the methods section of the manuscript (lines 516-519). To choose the number of PCR cycles for library preparation prior to sequencing, we performed a number of optimization tests which included using the same set of primers and running a qPCR experiment. Reviewer only Figure 2, shows a qPCR run in which matching stool, saliva and plasma samples belonging to three melanoma patients were amplified in duplicate. In addition, duplicate amplification of DNA extraction negative controls (DENCs) for plasma, along with non-template controls (NTCs) are shown. Based on these results, we determined that for plasma and DENCs, 30 cycles would be the minimum number of cycles required to obtain the necessary amount of PCR product for sequencing. However, for stool and saliva samples, the PCR reaction reached saturation at around 25 cycles and any additional cycles could potentially contribute to the generation and amplification of PCR errors and biases. We thus restricted these sample types to only 25 cycles of PCR prior to sequencing.*

6. How was the RMA-Aware PERMANOVA method implemented? How did it account for repeated measures?

*This test was implemented as an R function which was previously published by Lloyd-Price et al. Multi-omics of the gut microbial ecosystem in inflammatory bowel diseases. Nature. 2019;569:655–62. This omission has now been corrected in lines 611-615 of the manuscript.*

7. How was the FDR cutoff chosen for the limma differential abundance analyses? It seems like an effect size threshold may be a better choice for this purpose since the key goal is to filter possibly batch-linked ASVs, regardless of the number of samples, batches and ASVs (which would all have varying effects on the p-values and FDR).

We used the default 0.05 FDR cutoff for the identification of differential expression/abundance using limma. We agree that the number of samples, batches and ASVs included in a certain comparison will have a varying effect on the FDR. However, this is a general issue that applies to any multiple hypothesis testing procedure and can affect any study that reports on multiple comparisons.

*As suggested by the reviewer, we have also performed a decontamination procedure in which the differential abundance analysis by technical variables was filtered by an effect size threshold (i.e. fold-change of >2) using limma’s treat function. Instead of testing for ASVs that have true log-fold-changes different from zero, this function tests whether the true log2-fold-change is greater than a defined threshold. In this modified analysis, an ASV was considered to have a batch effect only when the difference between groups was more than double. Through this more permissive decontamination procedure, we identified a total of 56 “decontaminated ASVs”, an increase of 18 new ASVs compared to the original list (Reviewer only Figure 3). Importantly, all ASVs from the original list were retained. Whilst, this list is larger and could represent additional “genuine” ASVs, we have chosen to report the more stringent list of ASVs as set by the FDR cutoff. However, if the reviewer feels strongly, the list based on an effect size threshold can also be included in a revised version of the manuscript.*

8. How was the Faecalibacterium ASV selected for ddPCR validation? The discussion should mention any factors that might influence whether this result is likely to be generalizable to the other identified ASVs.

*We chose to validate the Faecalibacterium ASV specifically because it had an abundance near the average for all high-confidence ASVs identified. It is also a common gut bacterium present in some of the stool samples and thus it was of specific interest to verify this observation. Whilst we were not able to validate a large number of ASVs due to the fact that we had limited plasma DNA remaining after sequencing, we have been able to perform additional ddPCR validation of another similarly abundant “high-confidence ASV” (Clostridum sensu stricto 9 ) (updated Figure 4F; Sequencing vs ddPCR kappa = 0.69 (p-value < 0.001); Spearman’s Rho = 0.72 (p-value < 0.001)). We have also included a comment in the results to indicate that both of the ASVs chosen for validation were representative of ASVs that showed average abundance in our final list of high-confidence ASVs.*

9. Were the high-confidence ASVs identified here similar in taxonomy or other properties to the taxa identified by Poore et al in their metagenomic study?

*We compared the taxonomies of the ASVs that passed all of our bioinformatics decontamination framework against the decontaminated plasma taxa classified using Shogun from Poore et. al. The taxonomies of 18/33 (55%) of the high-confidence ASVs obtained from our initial [31 ASVs] and new extension cohorts [2 ASVs] were also reported by Poore et. al. We have included statements reporting the results of this comparison in lines 265-268, 339-341 and 355-359. The results of this comparison are also now shown in a new column added to Additional file2: Table S3 and Additional file 3: Table S7.*

**Second round of review**

**Reviewer 2**

Thanks to the authors for their efforts to address my comments. The new data strengthens the manuscript considerably and the Methods are much clearer. I don't think it is necessary to include the reviewer figures in the manuscript; however, it would be nice to add a sentence to the Discussion noting that future analysis of additional data and/or simulations could help refine the in silico decontamination criteria and parameters to further optimize accuracy and sensitivity.

**Authors Response**

**Point-by-point responses to the reviewers’ comments:**

*We thank the reviewer for their positive comments and added a sentence to the discussion noting that future analysis of additional data and/or simulations could help refine the in silico decontamination criteria and parameters to further optimize accuracy and sensitivity.*
